# cGAS/STING signalling in macrophages aggravates obliterative bronchiolitis via an IFN‐α‐dependent mechanism after orthotopic tracheal transplantation in mice

**DOI:** 10.1002/ctm2.70323

**Published:** 2025-04-28

**Authors:** Junhao Wan, Hao Liu, Chuangyan Wu, Ting Zhou, Fengjing Yang, Xiaoyue Xiao, Song Tong, Sihua Wang

**Affiliations:** ^1^ Department of Thoracic Surgery, Union Hospital, Tongji Medical College Huazhong University of Science and Technology Wuhan China; ^2^ Department of Vascular Surgery, Traditional Chinese and Western Medicine Hospital of Wuhan, Tongji Medical College Huazhong University of Science and Technology Wuhan China; ^3^ Department of Critical Care Medicine, Union Hospital, Tongji Medical College Huazhong University of Science and Technology Wuhan China; ^4^ Department of Thoracic Surgery, Xiangya Hospital Central South University Changsha China

**Keywords:** cGAS/STING signalling pathway, CTLA4‐Ig, obliterative bronchiolitis, type I interferon

## Abstract

**Background:**

Our previous findings have underscored the role of innate immunity in obliterative bronchiolitis (OB). However, despite the central importance of the cyclic GMP‒AMP synthase (cGAS)/stimulator of interferon genes (STING) signalling pathway in innate immune responses, its specific contribution to OB progression remains largely unexplored.

**Methods:**

A murine orthotopic tracheal transplantation model was established to replicate OB pathogenesis. RNA sequencing and single‐cell RNA sequencing data were analysed to investigate mechanisms underlying OB. Key molecules of the cGAS/STING pathway were assessed using immunofluorescence staining. Macrophage‐specific *Sting1* knockout mice were generated to investigate the role of the cGAS/STING pathway in OB. Haematoxylin and eosin staining and Masson's trichrome staining were utilised to evaluate allograft stenosis and fibrosis. Immune cell infiltration and cytokine expression were analysed using immunofluorescence staining and qRT‐PCR. Flow cytometry was used to characterise splenic T‐cell subsets and assess co‐stimulatory molecule expression in macrophages.

**Results:**

The cGAS/STING pathway was upregulated in macrophages infiltrating allografts. Macrophage‐specific *Sting1* knockout significantly attenuated alloreactive T‐cell responses and alleviated OB. Furthermore, *Sting1* deletion reduced the expression of inflammatory marker NOS2, antigen‐presenting molecule MHC class II and co‐stimulatory molecules (CD80 and CD86) in macrophages. Mechanistically, *Sting1* knockout inhibited the production of interferon‐α2 (IFN‐α2), while the protective effect of macrophage‐specific *Sting* knockout was reversed by IFN‐α2 administration. Importantly, STING inhibition enhanced the allograft tolerance‐promoting effects of cytotoxic T‐lymphocyte‐associated antigen 4‐Ig (CTLA4‐Ig), leading to the preservation of the airway epithelium.

**Conclusions:**

Our study demonstrated that cGAS/STING signalling pathway exacerbated allograft rejection in an IFN‐α2‐dependent manner. These findings provide insights into potential novel strategies for prolonging allograft survival.

**Key points:**

- cGAS/STING signalling pathway was activated in macrophages infiltrating allografts.
- cGAS/STING signalling pathway in macrophages exacerbated allograft rejection, promoted antigen‐presenting ability of macrophages and enhanced alloreactive T‐cell responses in an IFN‐α2‐dependent manner.
- STING inhibition potentiated the therapeutic efficacy of CTLA4‐Ig in OB.

## Graphical abstract

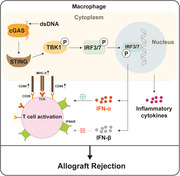

## 1 INTRODUCTION

Obliterative bronchiolitis (OB) represents a significant challenge to the long‐term survival of lung transplant recipients.^1^, ^2^ Immunosuppressive agents designed to inhibit alloreactive T cells in the adaptive immune system, such as cytotoxic T‐lymphocyte‐associated antigen 4‐Ig (CTLA4‐Ig), can partially slow OB progression.^3^ However, due to the involvement of innate immune cells, such as macrophages and neutrophils, these agents are unable to fully prevent OB progression.^4^, ^5^ For example, our team discovered that NACHT, LRR, and PYD domains‐containing protein (NLRP3) inflammasome and triggering receptor expressed on myeloid cells (TREM)‐1‐mediated innate immune signalling could aggravate OB progression.^6^, ^7^ Consequently, the development of novel therapeutic targets that specifically address the innate immune system has gained increasing attention.^8^

The cyclic GMP‒AMP synthase (cGAS)/stimulator of interferon genes (STING) signalling pathway, a cytosolic DNA sensing mechanism, could trigger a robust immune response by inducing the expression of type I interferons (IFN‐I), including IFN‐α and IFN‐β, as well as pro‐inflammatory cytokines in response to foreign or aberrant double‐stranded DNA (dsDNA).^9^, ^10^, ^11^ Dysregulation of this pathway has been implicated in autoimmune diseases such as systemic lupus erythematosus.^12^, ^13^ Moreover, cGAS/STING signalling is required for the activation of antigen‐presenting cells and the CD8^+^ T‐cell‐mediated antitumour immune response.^14^, ^15^, ^16^ However, research on the cGAS/STING pathway in transplant rejection remains limited,^17^ with only one recent study linking it to corneal allograft rejection.^18^ Therefore, investigating its role and potential mechanisms in OB is of significant importance.

This study demonstrated that the cGAS/STING pathway was activated in mice OB model. Inhibition of the cGAS/STING pathway limited the function of alloreactive T cells and resulted in reduced tracheal allograft stenosis and fibrosis. Mechanistically, macrophage‐specific *Sting1* ablation decreased IFN‐I production, while IFN‐α2 supplementation restored alloreactive T‐cell responses and exacerbated OB. Furthermore, blocking STING signalling in macrophages enhanced the immunosuppressive effects of CTLA4‐Ig on allograft rejection and promoted the preservation of ciliated columnar epithelium in the tracheal allografts.

## 2 MATERIALS AND METHODS

Details of the experimental materials and methods are provided in the Supporting Information (Tables S1 and S2).

### 2.1 Mice

Wild‐type (WT) C57BL/6J (B6) and BALB/c mice were provided by Vital River Laboratory Animal Technology Co., Ltd. *Sting1^fl^* (C57BL/6J‐*Sting1^em1(flox)Smoc^*) mice, *LysM^Cre^* (B6.129P2‐Lyz2^tm1(cre)Ifo^/J) mice and *Lysm^Cre^Sting1^fl^* B6 mice were generated in collaboration with Shanghai Model Organisms Center, Inc. All mice in this study were male, 8–10 weeks old, weighing 23–26 g, and housed in a specific pathogen‐free condition at Huazhong University of Science and Technology.

### 2.2 Orthotopic tracheal transplantation

The transplantation model was established following a published protocol from our laboratory.^6^, ^19^ The details are available in the Supporting Information.

### 2.3 Drug treatment

All dosages and schedules were based on published protocols. Clodronate liposomes or control liposomes (200 µL; YEASEN, Cat# 40337ES) were injected via the tail vein on days 1, 7, 14, and 21 after tracheal transplantation.^20^ The cGAS inhibitor RU.521 (5 mg/kg every 3 days; MedChemExpress, Cat# HY‐114180) was administered intraperitoneally (i.p.) for 21 days post‐transplantation.^18^ RO8191 (30 mg/kg/day; MedChemExpress, Cat# HY‐W063968), an agonist of interferon‐α/β receptor (IFNAR), was administered orally for 14 days post‐transplantation.^21^ Recombinant mouse IFN‐α2 (rIFN‐α2, 400 U/g; R&D Systems, Cat# 10149‐IF) and IFN‐β (rIFN‐β, 500–2000 U/day; Sigma, Cat# IF011) were injected i.p. on days 1, 3, 7, 14, and 21 post‐transplant.^22^, ^23^ The IFNAR antagonist antibody (anti‐IFNAR1, 250 µg; Selleck, A2460) was administered i.p. on days 1, 3, 7, 14, and 21.^24^ CTLA4‐Ig (200 µg; BioXCell, Cat# BE0099) was injected i.p. on days 0 and 2.^10^ The STING inhibitor C‐176 (750 nmol every 2 days; MedChemExpress, Cat# HY‐112906) was administered i.p. for 21 days.^25^

### 2.4 Histology assessment

Histological evaluation methods followed our previous work.^19^, ^26^ The details are described in the Supporting Information.

### 2.5 RNA sequencing, single‐cell RNA sequencing and bioinformatics analysis

RNA sequencing sampling was performed following the protocol described in *Eur Respir J. 2021;57(3):2000344* (PMID: 33033147).^27^ Bioinformatics analysis was conducted based on our previously published methods.^28^ Detailed descriptions can be found in the Supporting Information.

### 2.6 Statistics

All statistical data are provided in Table S3. *p*‐Values were calculated using GraphPad (version 10.0.3). Data are presented as mean ± standard deviation (SD) or mean ± standard error of the Mean (SEM). Statistical analysis between two independent groups was conducted using an unpaired Student's *t*‐test. For comparisons among multiple groups, one‐ or two‐way ANOVA was employed, followed by a least significant difference *t* post hoc test. When data did not meet the assumptions for parametric tests, the Mann‒Whitney *U*‐test was utilised. A *p*‐value of <.05 was considered statistically significant.

## 3 RESULTS

### 3.1 cGAS/STING pathway was activated in allograft macrophages

A murine orthotopic tracheal transplantation model was developed to replicate OB pathogenesis. Based previous studies and our team's earlier findings, allograft rejection peaked at day 7 post‐transplant, making it the optimal time point for assessing immune infiltration.^6^, ^7^, ^29^ Additionally, tracheal allograft fibrosis stabilised by day 28, marking it as the ideal time for evaluating stenosis and fibrosis.^29^

To explore mechanisms underlying allograft rejection, RNA sequencing was performed on syngrafts and allografts harvested 7 days post‐transplant (Figure S1A). Differentially expressed genes analysis identified 1005 upregulated and 1257 downregulated genes in allografts compared to syngrafts (Figure S1B). Consistent with previous findings,^8^, ^30^, ^31^ pathways known to promote allograft rejection, such as the Toll‐like receptor signalling pathway, were upregulated in allografts (Figure S1C). Notably, significant activation was observed in pathways related to DNA damage, particularly the cytosolic DNA sensing pathway and the RIG‐I‐like receptor (RLR) signalling pathway, which are closely associated with innate immunity (Figure S1D,E). Key genes in the cytosolic DNA sensing pathway (*Cgas*, *Sting*, *Tbk1*, *Irf7*, *Ifna2* and *Ifnb1*) and RLR pathway (*Ddx58* and *Mavs*) (Figures S1F and S2B) were markedly upregulated. This coordinated activation pattern implicated a possible involvement of the cGAS/STING signalling in allograft rejection. To further confirm the expression localisation of this pathway, we analysed a single‐cell dataset (GSE160760) of tracheal grafts published by Di Campli et al.^27^ Six major cell types were identified based on cell‐specific marker genes, with macrophages emerging as the predominant population in allografts (Figure S1G,H). Subsequent analysis of *Sting* expression across multiple cell types revealed the highest expression levels in macrophages (Figure S1I). Furthermore, *Sting* expression was significantly upregulated in allograft macrophages compared to their syngraft counterparts (Figure S1J).

**FIGURE 1:**
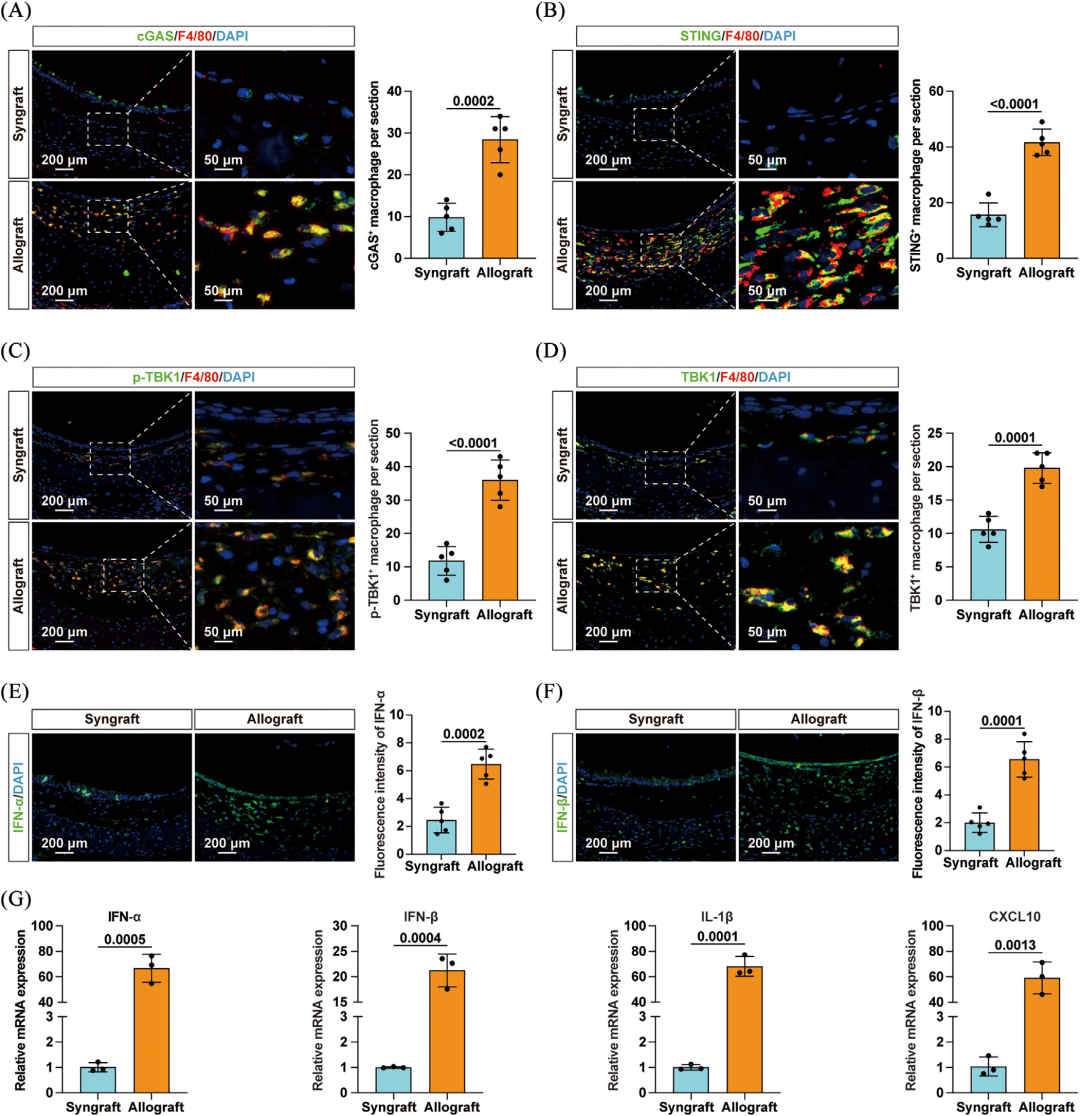
Cyclic GMP‒AMP synthase (cGAS)/stimulator of interferon genes (STING) pathway was upregulated in macrophages infiltrated in tracheal allografts. Representative immunofluorescence images (left panel) and corresponding statistical plots (right panel) showing (A) cGAS^+^ macrophages, (B) STING^+^ macrophages, (C) p‐TBK1^+^ macrophages and (D) TBK1^+^ macrophages infiltrating grafts collected on day 7 (*n* = 5 per group, mean ± SD, Student's *t*‐test). Representative immunofluorescence sections (left panel) and quantification charts (right panel) showing (E) interferon‐α (IFN‐α) and (F) IFN‐β in grafts (*n* = 5 per group, mean ± SD, Student's *t*‐test). (G) mRNA levels of IFN‐α, IFN‐β, interleukin‐1β (IL‐1β) and CXCL10 in grafts were measured by qPCR on day 7 (*n* = 3 per group, mean ± SD, Student's *t*‐test).

These findings were further supported by immunofluorescence staining, which revealed increased levels of cytosolic DNA (Figure S2A), as well as elevated expression of cGAS and STING (Figure 1A,B) in macrophages infiltrating the allografts. Additionally, Figure S2C‒E shows a pronounced increase in RIG‐I and MAVS protein expression in allograft‐infiltrating macrophages compared to syngrafts, while MDA5 levels remained statistically unchanged. Furthermore, there was an increased infiltration of p‐TBK1^+^ and TBK1^+^ macrophages in the allografts (Figure 1C,D). Elevated levels of IFN‐α and IFN‐β were also observed in allografts (Figure 1E,F). Correspondingly, mRNA levels of downstream cytokines, including IFN‐α, IFN‐β, interleukin‐1β (IL‐1β) and CXCL10, were significantly upregulated (Figure 1G). Taken together, these data demonstrated the activation of the cGAS/STING pathway and the RIG‐I/MAVS axis in macrophages infiltrating the allografts, suggesting their potential involvement in allograft rejection.

### 3.2 Inhibition of the cGAS/STING pathway ameliorated OB

**FIGURE 2:**
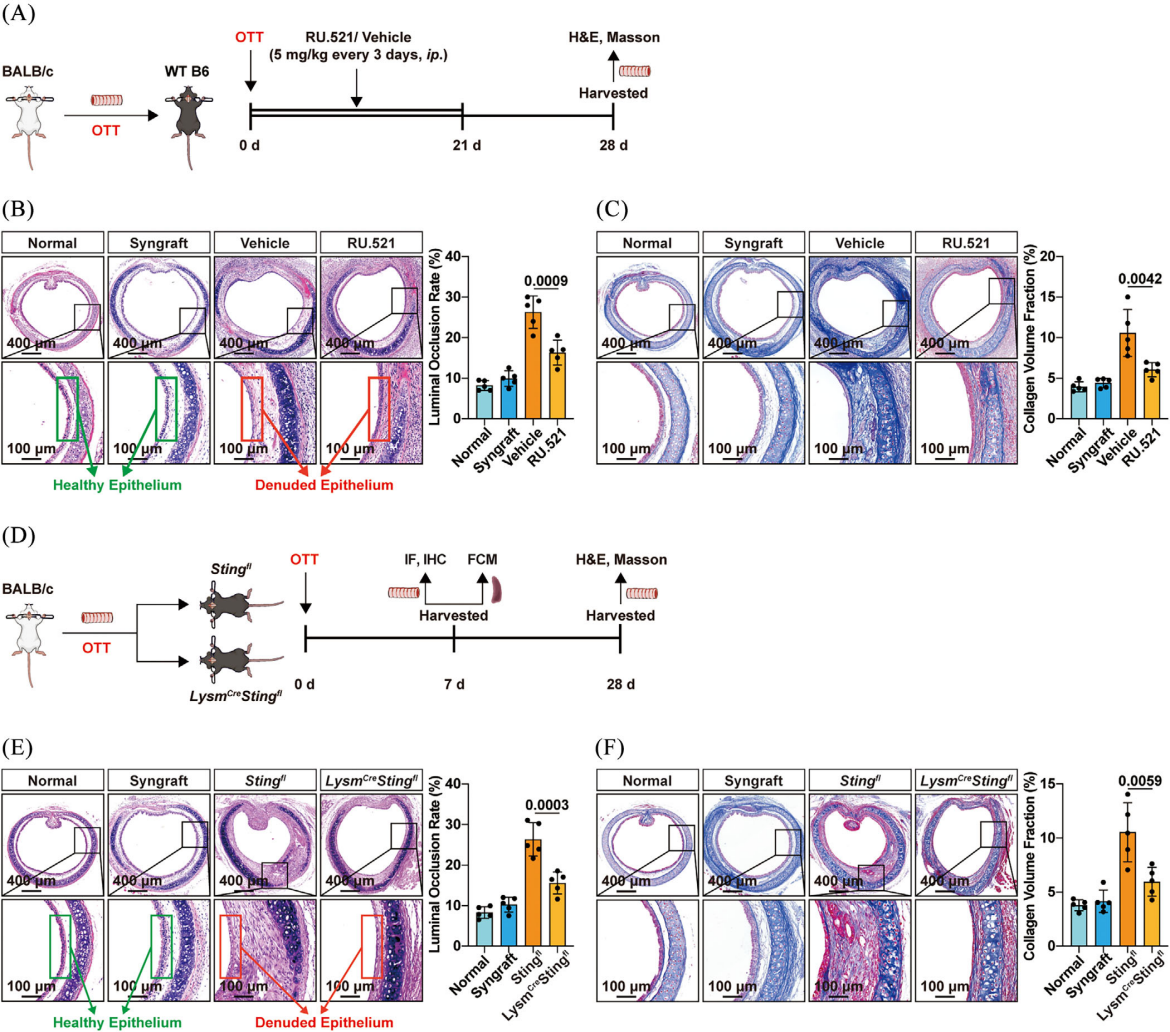
Inhibition of cyclic GMP‒AMP synthase (cGAS)/stimulator of interferon genes (STING) pathway alleviated obliterative bronchiolitis (OB). (A) Experiment workflow: recipient mice were intraperitoneally injected with cGAS inhibitor RU.521 to explore the role of the cGAS in OB. (B) Representative haematoxylin and eosin (H&E)‐stained sections of transplanted trachea collected on day 28 post‐transplant (left panel) and a statistical diagram (right panel) showing the degree of stenosis in tracheal allografts (*n* = 5 per group, mean  ± SD, two‐way ANOVA test). (C) Representative Masson staining sections of allografts obtained on day 28 (left panel) and statistical charts (right panel) illustrating collagen deposition (*n* = 5 per group, mean  ± SD, two‐way ANOVA test). (D) Experiment workflow: macrophage‐specific *Sting1* knockout mice were generated to explore the role of the cGAS/STING pathway in OB. (E) Representative H&E‐stained sections of transplanted trachea collected on day 28 post‐transplant (left panel) and a statistical diagram (right panel) showing the degree of stenosis in tracheal allografts (*n* = 5 per group, mean  ± SD, two‐way ANOVA test). (F) Representative Masson staining sections of allografts obtained on day 28 (left panel) and statistical charts (right panel) illustrating collagen deposition (*n* = 5 per group, mean ± SD, two‐way ANOVA test).

Next, recipient mice were injected with clodronate liposomes to investigate the role of macrophage depletion in the development of OB (Figure S3A). As shown in Figure S3B, mice treated with clodronate liposomes exhibited a significantly reduced luminal occlusion rate in tracheal allografts. Moreover, a marked reduction in fibrosis was observed within the allografts of clodronate liposome‐treated mice (Figure S3C). Subsequently, the cGAS inhibitor RU.521 and the STING inhibitor C‐176 were administered to elucidate the involvement of cGAS/STING signalling in OB. Pharmacological inhibition of cGAS with RU.521 alleviated tracheal allograft stenosis and fibrosis (Figure 2A–C). Similarly, treatment with C‐176 also ameliorated OB (Figure 6J–L). To determine whether the cGAS/STING pathway in macrophages contributed to OB, macrophage‐specific *Sting1* knockout (*Lysm^Cre^Sting1^fl^*) mice were generated as recipients (Figures 2D and S4A‒C). Compared to *Sting1^fl^* control mice, *Lysm^Cre^Sting1^fl^* mice exhibited significantly reduced tracheal allograft stenosis and fibrosis (Figure 2E,F). Unfortunately, similar to studies on other pathways,^3^ blocking the cGAS/STING signalling failed to protect the airway's ciliated columnar epithelial cells (0/5), ultimately leading to epithelial shedding in the allografts (Figure 2E). In summary, these findings revealed the pathogenic effect of the cGAS/STING pathway in OB progression.

### 3.3 *Sting1* deficiency in macrophages limited alloreactive T‐cell responses

**FIGURE 3:**
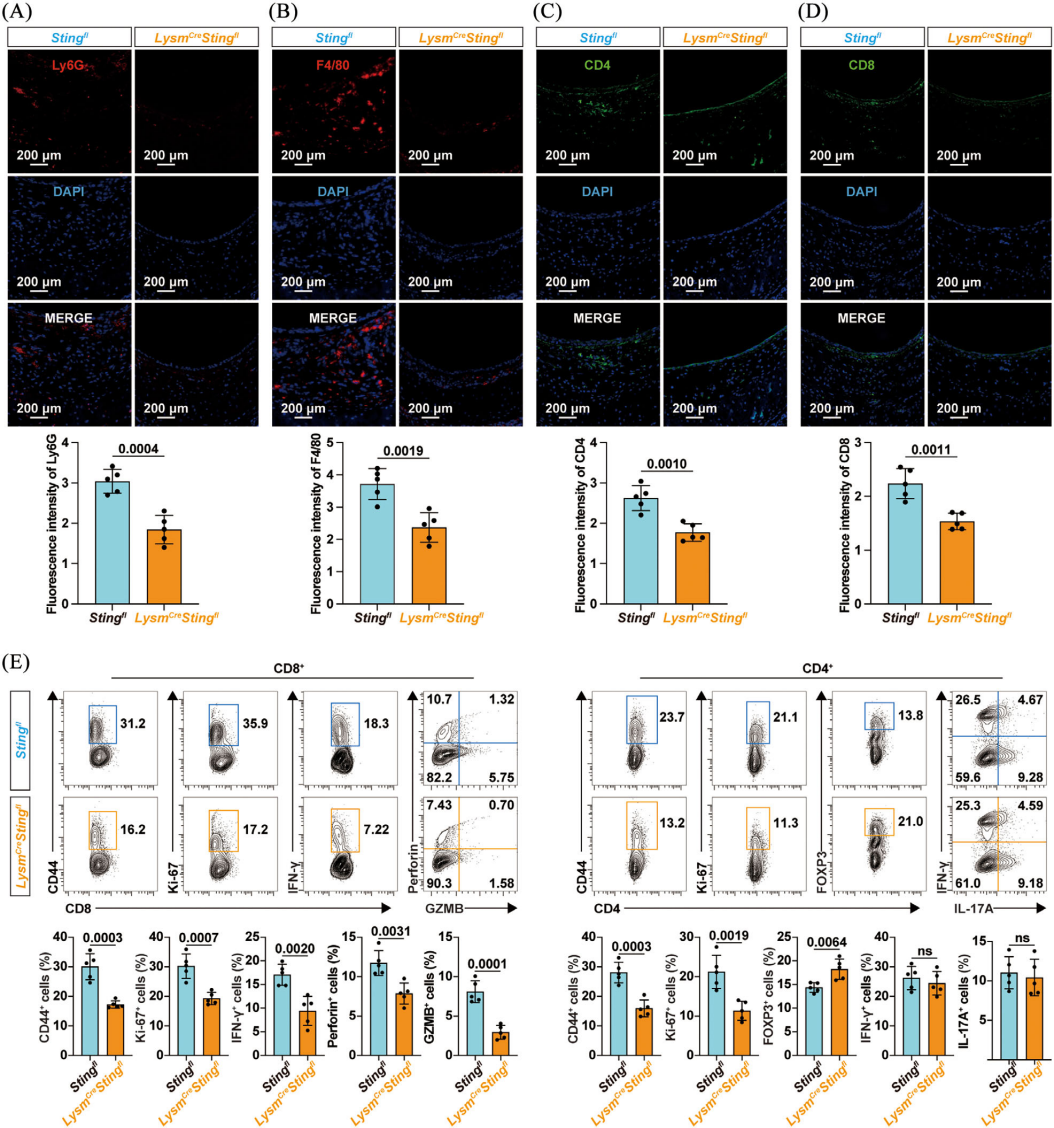
Macrophage‐specific *Sting* knockout limited alloreactive T‐cell responses. Representative immunofluorescence sections (left panel) and corresponding statistical plots (right panel) showing infiltration of (A) neutrophils, (B) macrophages, (C) CD4^+^ T cells and (D) CD8^+^ T cells in grafts (*n* = 5 per group, mean ± SD, Student's *t* test). (E) Representative flow cytometry plots and quantification charts showing the proportion of CD44, Ki‐67, interferon‐γ (IFN‐γ), perforin and GZMB‐expressing cells in splenic CD8^+^ T cells, as well as the percentage of CD44, Ki‐67, FOXP3, IFN‐γ and interleukin‐17A (IL‐17A)‐expressing cells in splenic CD4^+^ T cells (*n* = 5 per group, mean ± SD, Student's *t*‐test).

To comprehensively evaluate the influence of the cGAS/STING pathway in macrophages on allograft rejection, immune cell infiltration was assessed by immunofluorescence staining. *Sting1* deficiency in macrophages resulted in reduced infiltration of neutrophils and macrophages in the allografts (Figure 3A,B). *Lysm^Cre^Sting1^fl^* mice also exhibited decreased CD4^+^ and CD8^+^ T cells in tracheal allografts (Figure 3C,D). Given that T‐cell‐mediated adaptive immune responses are key determinants of allograft rejection, we characterised the molecular and functional phenotypes of splenic T cells on day 7 post‐transplant (Figures 3E and S5A). Flow cytometry revealed lower proportions of cells expressing the antigen‐experienced marker CD44 or the proliferation marker Ki‐67 in both CD8^+^ and CD4^+^ T cells in *Lysm^Cre^Sting1^fl^* mice (Figure 3E). Moreover, *Sting1* ablation in macrophages reduced the proportion of cells expressing effector molecules (IFN‐γ, perforin and granzyme B [GZMB]) in splenic CD8^+^ T cells. Interestingly, while the proportion of cells expressing IFN‐γ or IL‐17A in CD4^+^ T cells remained unchanged, there was a notable increase in FOXP3‐expressing CD4^+^ T cells (Tregs) in *Lysm^Cre^Sting1^fl^* mice. These data suggested that *Sting1* deletion in macrophages limited alloantigen‐reactive T‐cell responses, thereby contributing to the attenuation of transplant rejection.

### 3.4 *Sting* ablation inhibited macrophage IFN‐I production and antigen presentation

**FIGURE 4:**
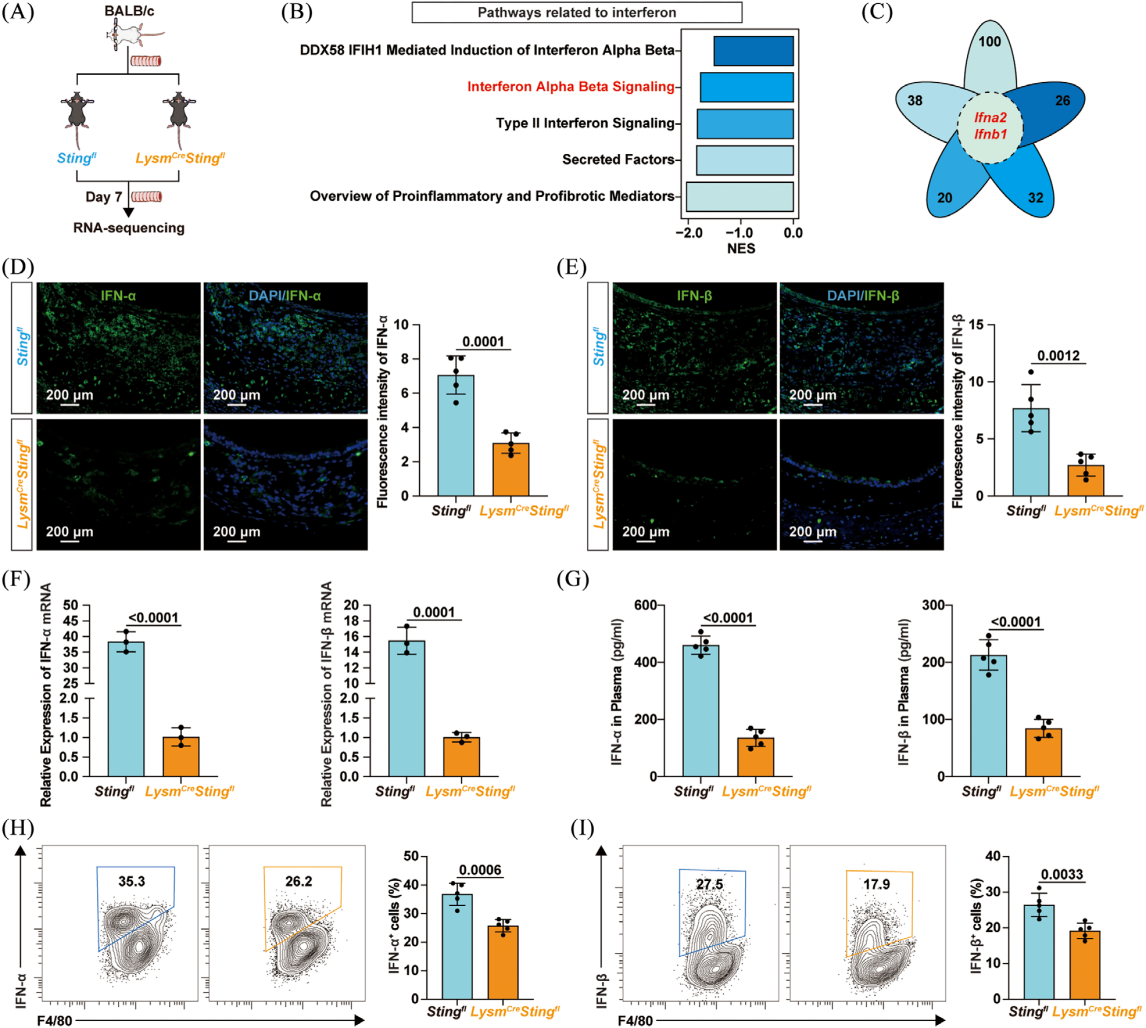
Macrophage‐specific *Sting* knockout inhibited type I interferon (IFN‐I) production. (A) Study design: allografts collected from *Sting1^fl^* and *Lysm^Cre^Sting1^fl^* mice on day 7 post‐transplant were used for RNA sequencing. (B) Pathways related to IFN were inhibited in macrophage‐specific *Sting* knockout mice. (C) Petal diagram showing the intersection genes of IFN‐related signalling pathways. Representative immunofluorescence sections (left panel) and corresponding statistical plots (right panel) showing (D) IFN‐α and (E) IFN‐β infiltration in allografts (*n* = 5 per group, mean ± SD, Student's *t*‐test). (F) mRNA levels of IFN‐α (left panel) and IFN‐β (right panel) in allografts (*n* = 3 per group, mean ± SD, Student's *t*‐test). (G) ELISA showing IFN‐α (left panel) and IFN‐β (right panel) levels in plasma collected on day 7 (*n* = 5 per group, mean ± SD, Student's *t*‐test). Representative flow cytometry plots (left panel) and statistical charts (right panel) showing the proportion of (H) IFN‐α and (I) IFN‐β‐expressing cells in macrophages (*n* = 5 per group, mean ± SD, Student's *t*‐test).

To clarify how the cGAS/STING pathway in macrophages contributed to allograft rejection and OB exacerbation, allografts from *Sting1^fl^* mice and *Lysm^Cre^Sting1^fl^* mice were harvested for RNA sequencing (Figure 4A). GSEA revealed that pathways related to classical cytokines, such as the transforming growth factor‐beta (TGF‐β) signalling pathway, were inhibited by macrophage‐specific *Sting* knockout (Figure S6A). Pathways involved in the IFN response, such as the DDX58/IFIH1‐mediated induction of IFN‐α/β pathway, were also downregulated in *Lysm^Cre^Sting1^fl^* mice (Figure 4B). Heatmap in Figure S7A shows that *Sting1* ablation led to reduced mRNA expression of RIG‐I, MDA5 and MAVS. Immunofluorescence staining further revealed decreased expression levels of RIG‐I and MAVS in macrophages within allografts from *Lysm^Cre^Sting1^fl^* mice, whereas MDA5 expression showed no statistically significant difference (Figure S7B‒D). These findings suggested that macrophage‐specific *Sting1* deletion suppressed activation of the RIG‐I/MAVS pathway in allografts. Notably, the petal diagram in Figure 4C shows that *Ifna2* and *Ifnb1* were both enriched and downregulated in these IFN‐related pathways. Experimental results further confirmed that *Sting1* ablation in macrophages reduced the infiltration of IFN‐α and IFN‐β in allografts at both the protein and mRNA levels (Figure 4D‒F). Plasma levels of IFN‐α and IFN‐β were also lower in *Lysm^Cre^Sting1^fl^* mice (Figure 4G). Moreover, macrophage‐specific *Sting1* knockout reduced the proportion of cells producing IFN‐α and IFN‐β within macrophages (Figures 4H,I and S8A). Overall, *Sting1* deficiency inhibited IFN‐I production in macrophages.

Macrophages not only secrete cytokines that directly damage allografts, but also act as antigen‐presenting cells to promote alloreactive T‐cell responses.^32^, ^33^ GSEA revealed that the MHC class II antigen presentation pathway, reflecting antigen‐presenting function, was downregulated in allografts from *Lysm^Cre^Sting1^fl^* mice (Figure S6B). As shown in Figure S6C, mRNA levels of the pro‐inflammatory marker NOS2 and co‐stimulatory molecules CD80 and CD86 were also reduced. Flow cytometry further demonstrated that *Sting1* knockout decreased the expression of NOS2 (Figure S6D), MHC class II (Figure S6E) and co‐stimulatory molecules (CD80 and CD86) in macrophages (Figure S6F,G), indicating a diminished antigen‐presenting capacity. Collectively, these findings suggested that *Sting1* deletion reduced IFN‐I production and impaired the antigen‐presenting function of macrophages.

### 3.5 rIFN‐α2 treatment aggravated OB in *Lysm^Cre^Sting1^fl^* mice

**FIGURE 5:**
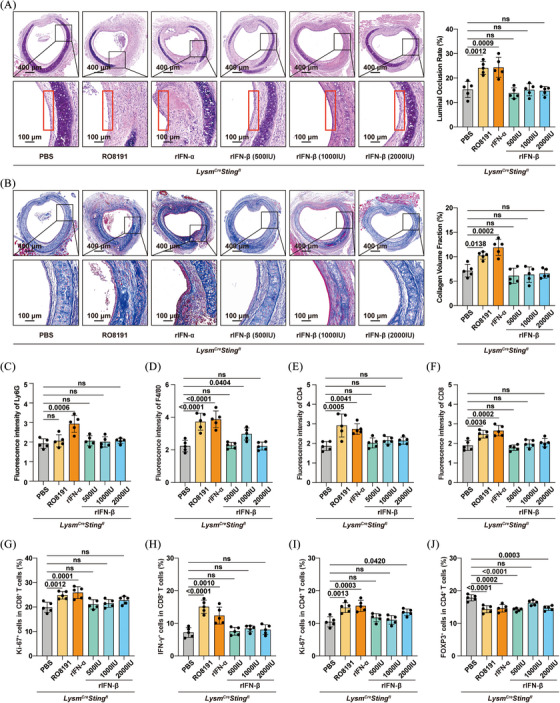
Interferon‐α (IFN‐α) treatment exaggerated obliterative bronchiolitis (OB) in *Lysm^Cre^Sting1^fl^* mice. (A) Representative haematoxylin and eosin (H&E) sections of tracheal allografts harvested on day 28 (left panel) and corresponding statistical diagram (right panel) showing the degree of stenosis (*n* = 5 per group, mean ± SD, two‐way ANOVA test). (B) Representative Masson staining sections of the transplanted trachea (left panel) and corresponding statistical charts (right panel) illustrating collagen deposition (*n* = 5 per group, mean ± SD, two‐way ANOVA test). Bar charts showing the mean fluorescence intensity of (C) Ly6G, (D) F4/80, (E) CD4 and (F) CD8 in allografts (*n* = 5 per group, mean ± SD, two‐way ANOVA test). Bar plots showing (G) Ki‐67 and (H) IFN‐γ‐expressing cell subsets within CD8^+^ T cells (*n* = 5 per group, mean ± SD, two‐way ANOVA test). Bar charts showing (I) Ki‐67 and (J) FOXP3 expression in CD4^+^ T cells (*n* = 5 per group, mean ± SD, two‐way ANOVA test).

To investigate the role of IFN‐I in OB, recipient mice were treated with an antagonist antibody targeting IFNAR1 (anti‐IFNAR1) (Figure S9A). The results demonstrated that anti‐IFNAR1 treatment significantly alleviated tracheal allograft stenosis and fibrosis, implicating the involvement of IFN‐I in allograft rejection (Figure S9B,C). Subsequently, to further assess whether the cGAS/STING pathway exacerbated allograft rejection via the IFN‐I axis, *Lysm^Cre^Sting1^fl^* mice were administered RO8191 (30 mg/kg/day for 14 days), an IFNAR agonist.^21^ As expected, RO8191 treatment aggravated stenosis and fibrosis of the transplanted trachea, with the airway epithelium remaining in a state of shedding (Figure 5A,B). Although no difference in neutrophil infiltration was observed (Figure 5C), the infiltration of macrophages, CD4^+^ T cells, and CD8^+^ T cells was increased in the RO8191‐treated *Lysm^Cre^Sting1^fl^* mice (Figure 5D‒F). Additionally, RO8191 elevated the proportion of Ki‐67^+^ cells within both CD4^+^ and CD8^+^ T‐cell populations (Figure 5G,H), as well as the percentage of CD8^+^ T cells expressing IFN‐γ (Figure 5I). Conversely, RO8191 decreased the proportion of Tregs within the CD4^+^ T‐cell population (Figure 5J). These findings suggest that IFN‐I contributes to allograft rejection as a downstream effector of the cGAS/STING pathway; however, it remains unclear whether IFN‐α or IFN‐β is responsible.

Therefore, we supplemented the *Lysm^Cre^Sting1^fl^* mice with either rIFN‐α2 (400 U/g/day) or rIFN‐β (500, 1000 or 2000 U/day) on days 1, 3, 7, 14 and 21 post‐transplant.^22^, ^23^ Intriguingly, rIFN‐α2 exacerbated OB, whereas rIFN‐β had no observable effect (Figure 5A,B). Correspondingly, rIFN‐α2 increased immune cell infiltration (neutrophils, macrophages and CD4^+^/CD8^+^ T cells) within the allografts (Figure 5C‒F), elevated the percentage of Ki‐67^+^ cells among both CD4^+^ and CD8^+^ T cells (Figure 5G,I), increased the proportion of IFN‐γ^+^ cells among splenic CD8^+^ T cells (Figure 5H), and reduced the proportion of Tregs within splenic CD4^+^ T cells (Figure 5J). Overall, these data indicated that IFN‐α2, rather than IFN‐β, was a key downstream effector through which the cGAS/STING pathway promoted allograft rejection.

### 3.6 STING inhibition enhanced the immunosuppressive effect of CTLA4‐Ig

**FIGURE 6:**
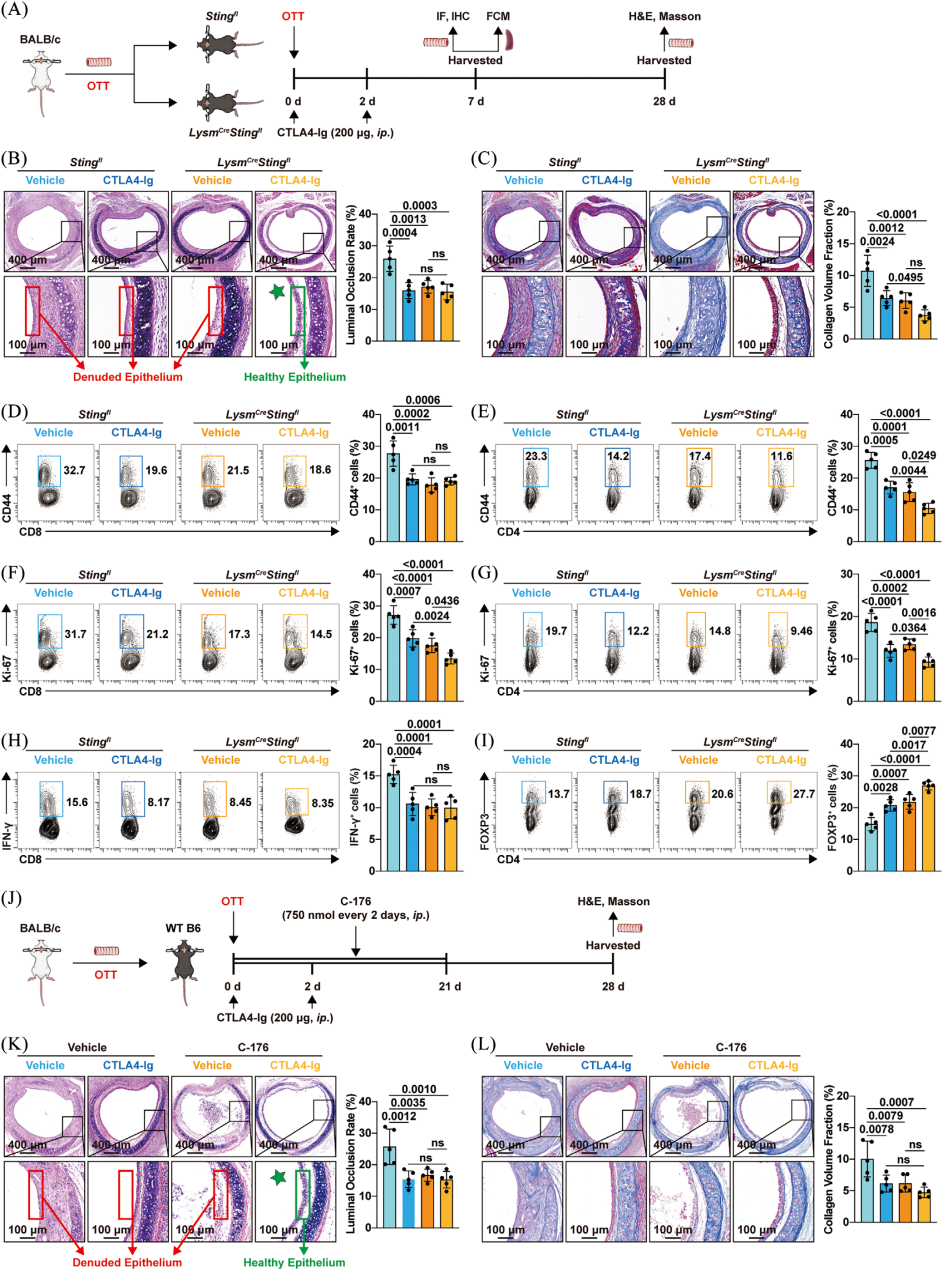
Stimulator of interferon genes (STING) inhibition enhanced the therapeutic effect of cytotoxic T‐lymphocyte‐associated antigen 4‐Ig (CTLA4‐Ig). (A) Schematic diagram of the experimental design: mice were treated with CTLA4‐Ig (200 µg/day) on days 0 and 2 post‐transplant. (B) Representative haematoxylin and eosin (H&E)‐stained sections of tracheal allografts (left panel) and corresponding statistical diagrams (right panel) showing the degree of stenosis in transplanted trachea (*n* = 5 per group, mean ± SD, two‐way ANOVA test). (C) Representative Masson's staining sections of transplanted trachea (left panel) and statistical diagrams (right panel) illustrating collagen deposition (*n* = 5 per group, mean ± SD, two‐way ANOVA test). Representative flow cytometry plots (left panel) and corresponding statistical charts (right panel) showing the proportion of (D and E) CD44 and (F and G) Ki‐67‐expressing cells in CD4^+^ T cells and CD8^+^ T cells (*n* = 5 per group, mean ± SD, two‐way ANOVA test). (H) Representative flow cytometry diagram (left panel) and statistical charts (right panel) showing the proportion of IFN‐γ‐expressing cells in CD8^+^ T cells (*n* = 5 per group, mean ± SD, two‐way ANOVA test). (I) Representative flow cytometry diagram (left panel) and statistical charts (right panel) showing the proportion of FOXP3‐expressing cells in CD4^+^ T cells (*n* = 5 per group, mean ± SD, two‐way ANOVA test). (J) Experiment workflow: recipient mice were intraperitoneally injected with C‐176 or CTLA4‐Ig. (K) Representative H&E‐stained sections of trachea (left panel) and a statistical diagram (right panel) showing the degree of stenosis in tracheal allografts (*n* = 5 per group, mean ± SD, two‐way ANOVA test). (L) Representative Masson staining sections of trachea (left panel) and statistical charts (right panel) illustrating collagen deposition (*n* = 5 per group, mean ± SD, two‐way ANOVA test).

CTLA4‐Ig has been approved by the Food and Drug Administration for immunosuppressive therapy in kidney transplantation.^34^ Previous studies have shown that CTLA4‐Ig could alleviate tracheal stenosis and fibrosis.^3^ However, it shares a crucial limitation with macrophage‐specific *Sting1* knockout: neither approach can protect the airway epithelium from damage. Considering combination therapy is an effective strategy to enhance treatment efficacy, and that FTY720 combined with CTLA4‐Ig have been reported to protect respiratory epithelium of tracheal allografts,^35^ we sought to determine whether combining STING inhibition with CTLA4‐Ig treatment could preserve the ciliated columnar epithelium. Thus, *Sting1^fl^* and *Lysm^Cre^Sting1^fl^* mice were injected with either CTLA4‐Ig or vehicle (200 µg/day) on days 0 and 2 (Figure 6A). Consistent with previous findings,^35^ CTLA4‐Ig alone reduced tracheal allograft stenosis and fibrosis but failed to preserve the ciliated columnar epithelium (Figure 6B,C). Remarkably, CTLA4‐Ig treatment in *Lysm^Cre^Sting1^fl^* mice not only mitigated airway stenosis and fibrosis but also preserved the ciliated columnar epithelium (4/5)—an effect not observed with either treatment alone (Figure 6B,C). Moreover, *Sting1* ablation enhanced the inhibitory effect of CTLA4‐Ig on alloreactive T‐cell responses. While there was no significant difference in memory CD8⁺ T cells between the combination group and CTLA4‐Ig alone (Figure 6D), combination therapy significantly reduced the proportion of memory CD4^+^ T cells (Figure 6E), as well as the Ki‐67^+^ population in both CD4^+^ and CD8^+^ T cells (Figure 6F,G). Furthermore, it led to a marked increase in Tregs among CD4^+^ T cells (Figure 6I), without significantly affecting effector CD8⁺ T cells (Figure 6H). Additionally, considering that pharmacological intervention is more clinically translatable than genetic knockout, we treated recipient mice with a combination of CTLA4‐Ig and the STING inhibitor C‐176 (Figure 6J), and found that this combination effectively protected the airway epithelium (4/5) (Figure 6K,L). Collectively, these results demonstrated that STING inhibition potentiated the therapeutic efficacy of CTLA4‐Ig in OB.

## 4 DISCUSSION

This study demonstrated the pathogenic role of the cGAS/STING pathway in allograft rejection using a murine OB model. Macrophage‐specific *Sting1* knockout inhibited IFN‐α2 production, thereby attenuating allograft rejection. Moreover, both genetic ablation and pharmacological inhibition of STING could enhance the therapeutic efficacy of CTLA4‐Ig in OB. Collectively, these findings underscore the cGAS/STING pathway as a promising therapeutic target for the treatment of OB.

As a cytosolic DNA sensor, cGAS could detect foreign microbial DNA and initiate antiviral and antibacterial immune responses.^36^, ^37^ cGAS could also recognise host‐derived dsDNA released into the cytosol under conditions of cellular stress or damage. Such dsDNA may originate from nuclear leakage via micronuclei rupture, chromosomal instability, mitotic errors or genotoxic stress, as well as from mitochondrial DNA released by dysfunctional mitochondria.^38^ These endogenous dsDNA could activate the cGAS/STING pathway, thereby enhancing the activity of antigen‐presenting cells and T cells, and contributing to antitumour immunity, autoimmune diseases and chronic inflammation.^11^, ^39^ In this study, we found that the cGAS/STING pathway was upregulated in macrophages infiltrating the allografts, and that macrophage‐specific *Sting1* knockout inhibited T‐cell memory formation, proliferation and effector functions. Similar to its role in exacerbating corneal allograft rejection,^18^ both pharmacological inhibition and genetic ablation of STING alleviated tracheal allografts stenosis and fibrosis. These findings support a pathogenic role for the cGAS/STING pathway in the development of OB.

IFN‐I, including IFN‐α and IFN‐β, are secreted upon activation of the cGAS/STING pathway.^40^, ^41^ These cytokines typically augmented immune responses by binding to their shared receptor on cell surfaces.^42^, ^43^ In this study, we found that both IFN‐α and IFN‐β were downregulated following *Sting* knockout. Administration of exogenous rIFN‐α2 to *Lysm^Cre^Sting1^fl^* mice exacerbated OB, which was consistent with previous research showing that IFN‐α blockade alleviated cardiac allograft rejection.^22^ In contrast, IFN‐β administration alone had no significant effect on transplant rejection, further confirming earlier findings.^10^ Overall, these results suggested that IFN‐α2, rather than IFN‐β, played pathogenic role in OB. Future studies showed further explore the contribution of other IFN‐α subtypes to allograft rejection.

Combination therapy represents an effective strategy for suppressing allograft rejection. In line with previous studies,^35^ genetic ablation or pharmacological inhibition of STING failed to protect the airway epithelium. However, combining STING inhibition with CTLA4‐Ig treatment significantly enhanced immunosuppressive efficacy and effectively preserved the airway epithelium. These results underscore the potential of cGAS/STING pathway inhibition as an adjunct to CTLA4‐Ig‐based therapy. However, additional studies are necessary to clarify the mechanisms by which this combination mediates epithelial protection.

To adequately replicate the physiological perfusion and complex surgical procedures of lung transplantation, the current findings should be further validated in a murine orthotopic lung transplant model.^44^ Moreover, the role of the cGAS/STING pathway in donor macrophages in the context of OB remains to be elucidated. And further research is required to investigate the source of dsDNA during allograft rejection. Finally, the expression of the cGAS/STING pathway and IFN‐I in patients with OB warrants additional clinical investigation.

In conclusion, this study revealed that the cGAS/STING pathway promoted allograft rejection and exacerbated OB in an IFN‐α2‐dependent manner. These findings may provide novel pharmacological targets for the treatment of OB following lung transplantation.

## AUTHOR CONTRIBUTIONS

Sihua Wang, Song Tong and Xiaoyue Xiao conceived and designed this study. Junhao Wan drafted the manuscript. Junhao Wan, Ting Zhou and Fengjing Yang performed the experiments. Junhao Wan and Song Tong analysed the data. All authors approved the final version of this manuscript.

## CONFLICT OF INTEREST STATEMENT

The authors declare they have no conflicts of interest.

## ETHICS STATEMENT

Animal experiments were conducted following the Guidelines for the Care and Use of Laboratory Animals (8th edition, National Institutes of Health) and approved by the Institutional Animal Care and Use Committee of Huazhong University of Science and Technology (protocol number 4043).

## Supporting information



Supporting information

## Data Availability

All the data are available from the corresponding author upon reasonable request. The raw sequence data reported in this paper have been deposited in the Genome Sequence Archive in National Genomics Data Center, China, National Center for Bioinformation/Beijing Institute of Genomics and Chinese Academy of Sciences (GSA: CRA019324) that are publicly accessible at https://ngdc.cncb.ac.cn/gsa.
