# Supplementary material for: cGAS/STING signalling in macrophages aggravates obliterative bronchiolitis via an IFN‐α‐dependent mechanism after orthotopic tracheal transplantation in mice
Source: Clin Transl Med. 2025 Apr 28;15(5):e70323. doi: 10.1002/ctm2.70323 (PMC12035648; doi:10.1002/ctm2.70323)
Supplement: Supplementary file 1 — Supporting information [file CTM2-15-e70323-s001.docx]

**Supplementary Material Content:**

# Supplementary Figures with Figure Legends

# Supplementary Materials and Methods

# Supplementary Tables

**Supplementary Figures with Figure Legends**

**
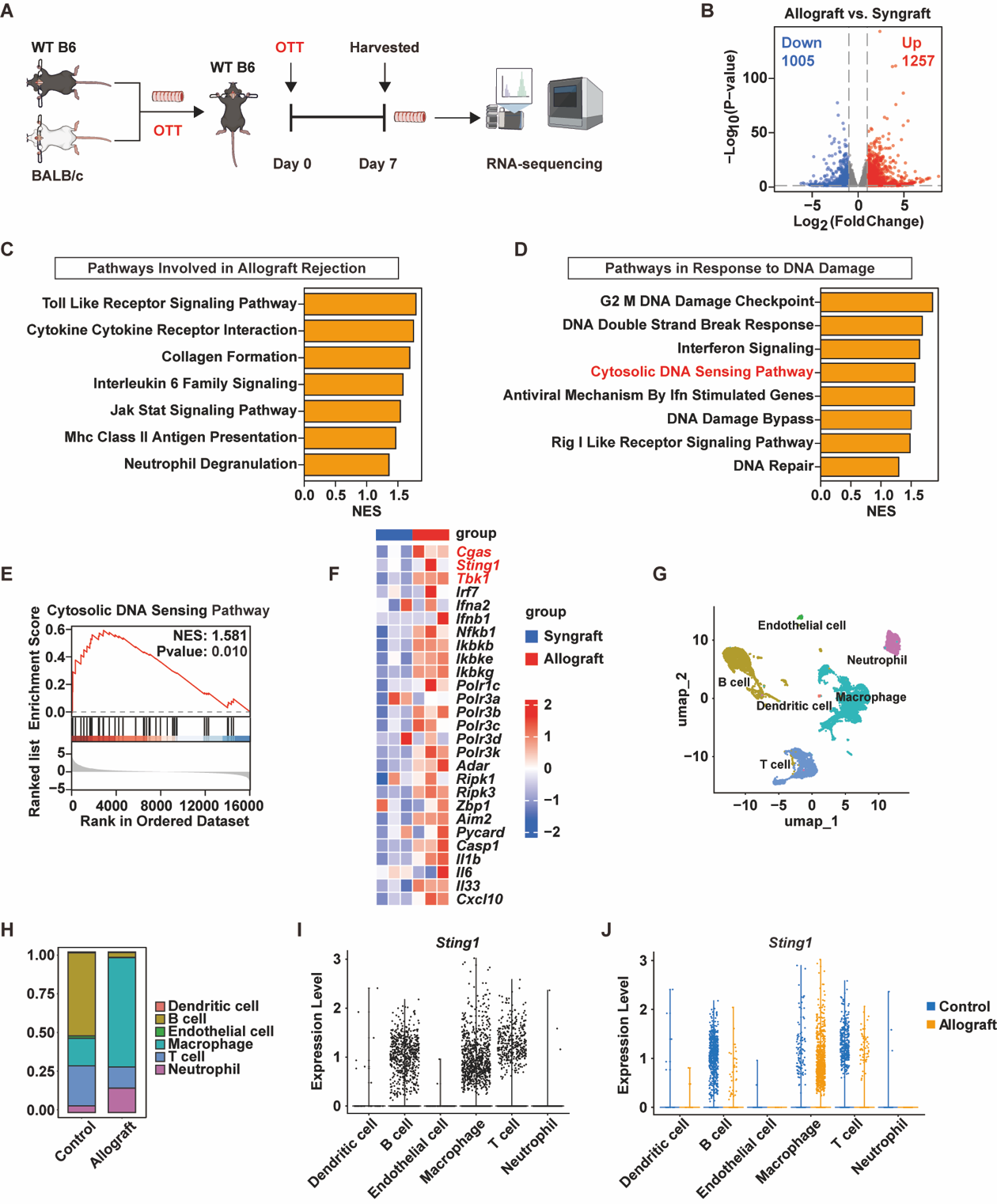
**

**Figure S1. Bioinformatic analysis revealed that cGAS/STING pathway was activated in macrophages within allografts (Related to Figure 1).**

1. Experiment design: Syngrafts and allografts harvested on day 7 post-transplant were used for RNA sequencing. In order to meet the requirements of sequencing, total RNA extracted from four grafts was combined as one sequencing sample.
2. Volcano plot showing DEGs identified by comparing allografts with syngrafts.
3. Pathways known to promote allograft rejection.
4. Pathways associated with DNA damage.
5. GSEA_KEGG showing the upregulated Cytosolic DNA Sensing Pathway.
6. Heatmap showing the genes enriched in the Cytosolic DNA Sensing Pathway.
7. ﻿Uniform Manifold Approximation and Projection (UMAP) plots showing the major cell clusters of grafts.
8. Bar plots showing the proportion of six cell types among different groups.
9. The expression level of *Sting1* in six cell clusters.
10. The expression level of *Sting1* in six cell types among different groups.

**
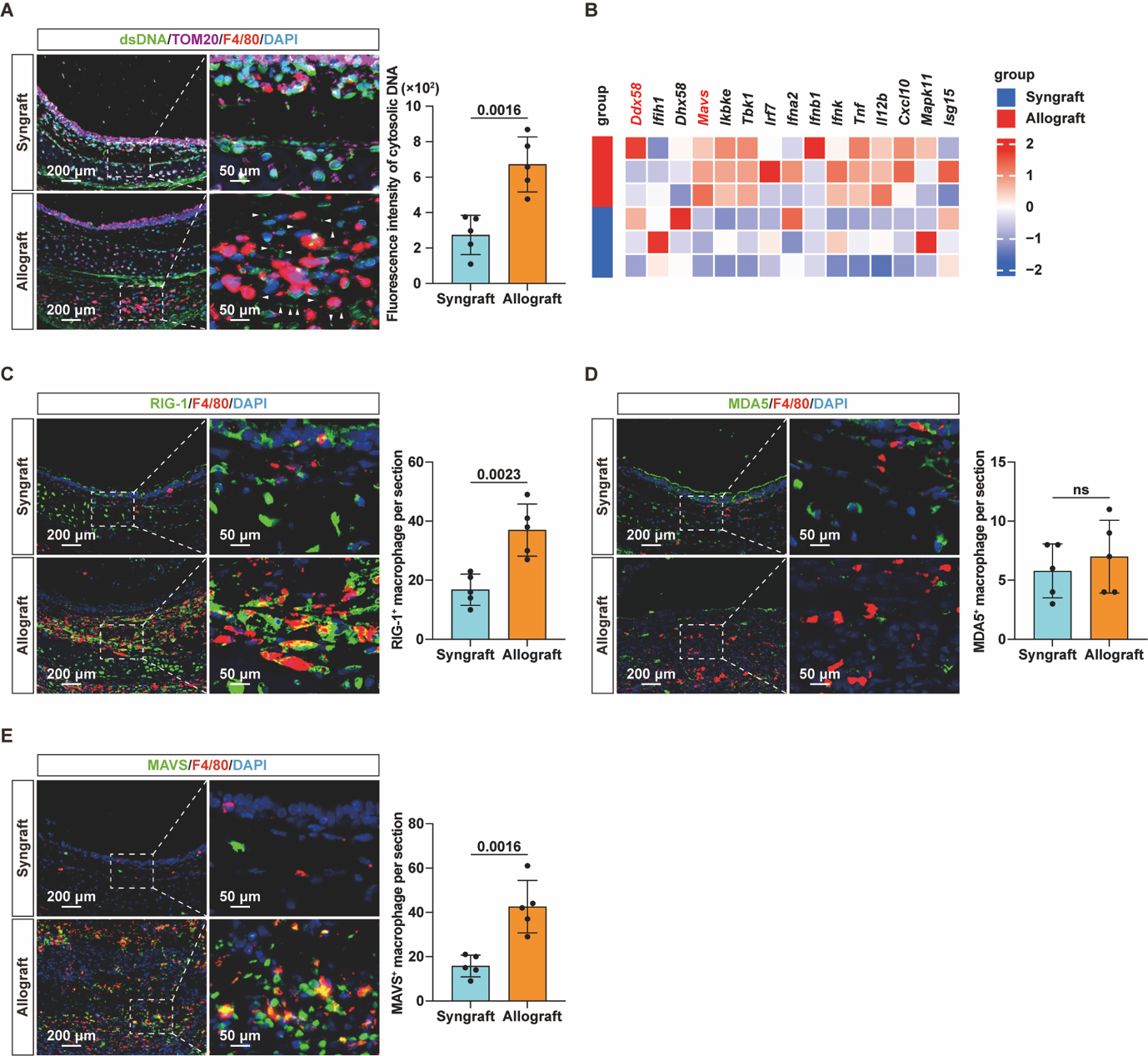
**

**Figure S2. The expression pattern of cytosolic DNA and RLR signaling pathway in grafts. (Related to Figure S1).**

1. Representative immunofluorescence images showing cytosolic DNA in macrophages infiltrating syngrafts and allografts (n = 5 per group, mean ± SD, Student’s *t* test).
2. Heatmap showing the genes enriched in the RLR signaling pathway.

(C-E) Representative immunofluorescence images showing (C) RIG-1, (D) MDA5, and (E) MAVS in macrophages infiltrating syngrafts and allografts (n = 5 per group, mean ± SD, Student’s *t* test).

**
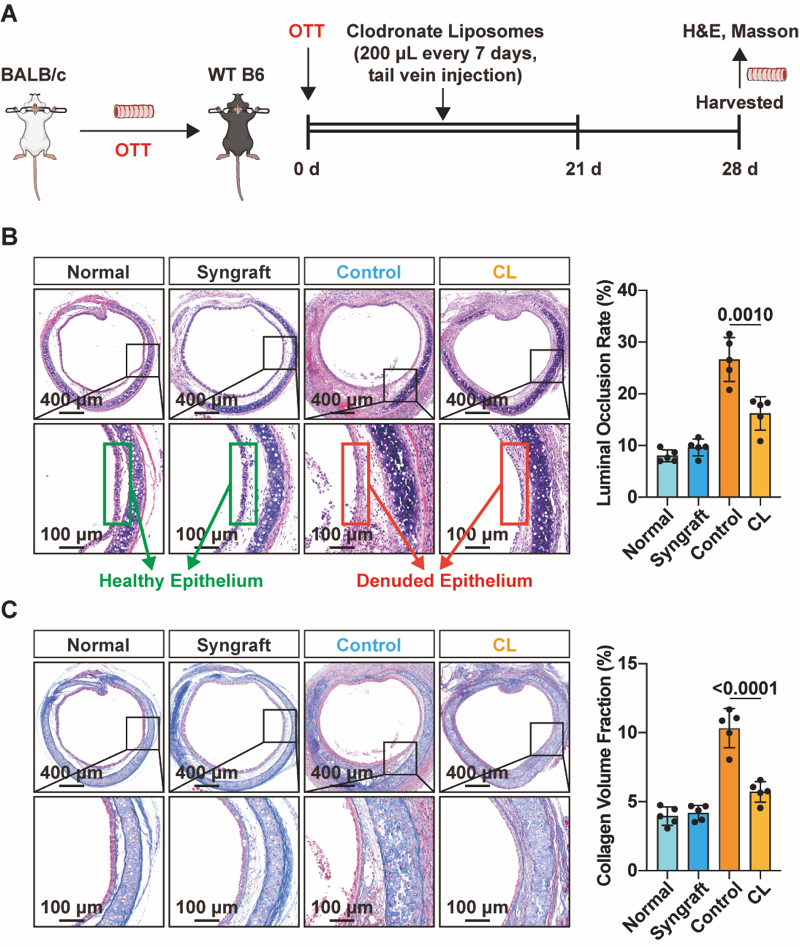
**

**Figure S3. Macrophage deletion alleviated OB**

(A) Experiment workflow: Recipient mice were injected with clodronate liposomes via the tail vein to investigate the role of macrophage clearance in OB.

(B) Representative H&E-stained sections of trachea (left panel) and a statistical diagram (right panel) showing the degree of stenosis in tracheal allografts. (n = 5 per group, mean ﻿± SD, two-way ANOVA test).

(C) Representative Masson staining sections of trachea (left panel) and statistical charts (right panel) illustrating collagen deposition (n = 5 per group, mean ﻿± SD, two-way ANOVA test).


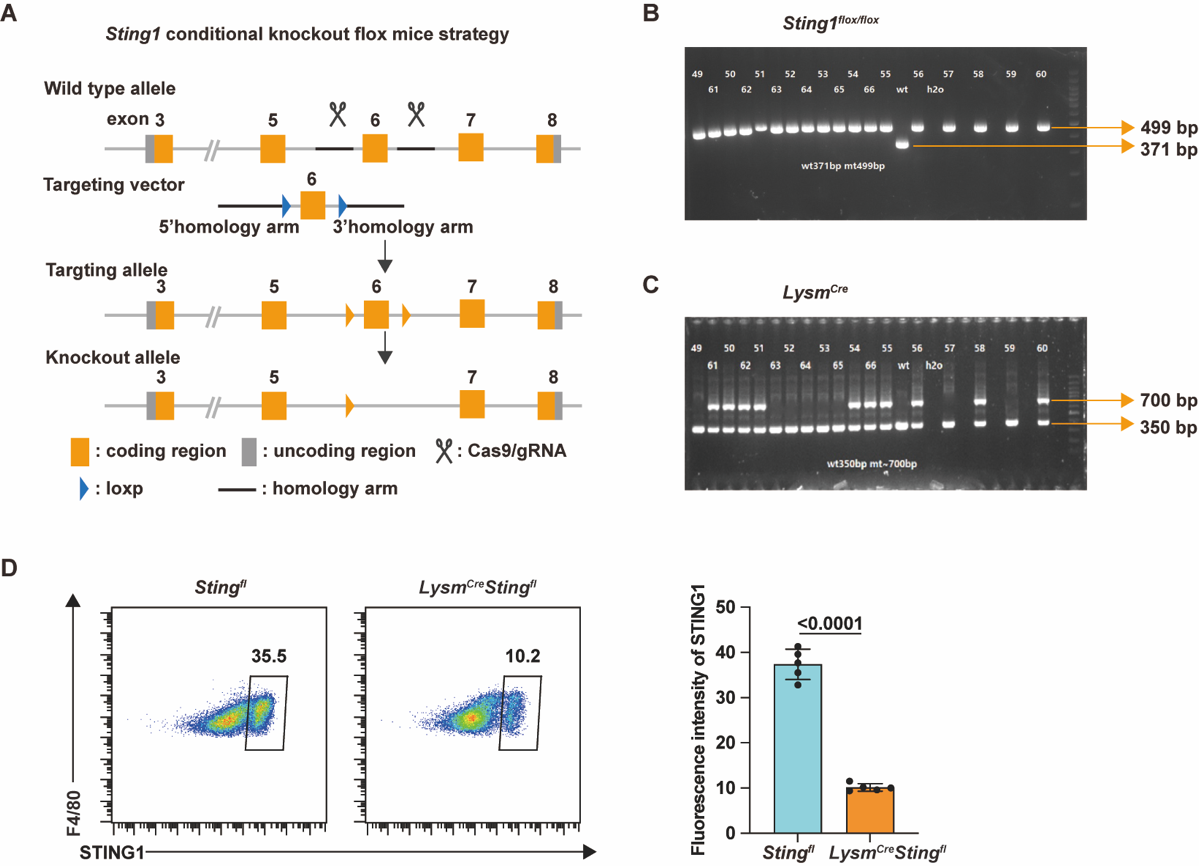


**Figure S4. Generation and identification of *Lysm^Cre^Sting1^fl^* mice (Related to** **Figure 2).**

1. Schematic diagram of the strategy used to generate *Sting* conditional knockout flox mice.

(B-C) Southern blot analysis identifying the expression of (B) *Sting1^flox/flox^* and (C) LysM-Cre in the DNA of different mice.

(D) Representative flow cytometry plots (left panel) and statistical charts (right panel) showing the proportion of STING expressing cells in macrophages (n = 5 per group, mean ± SD, Student’s *t* test).


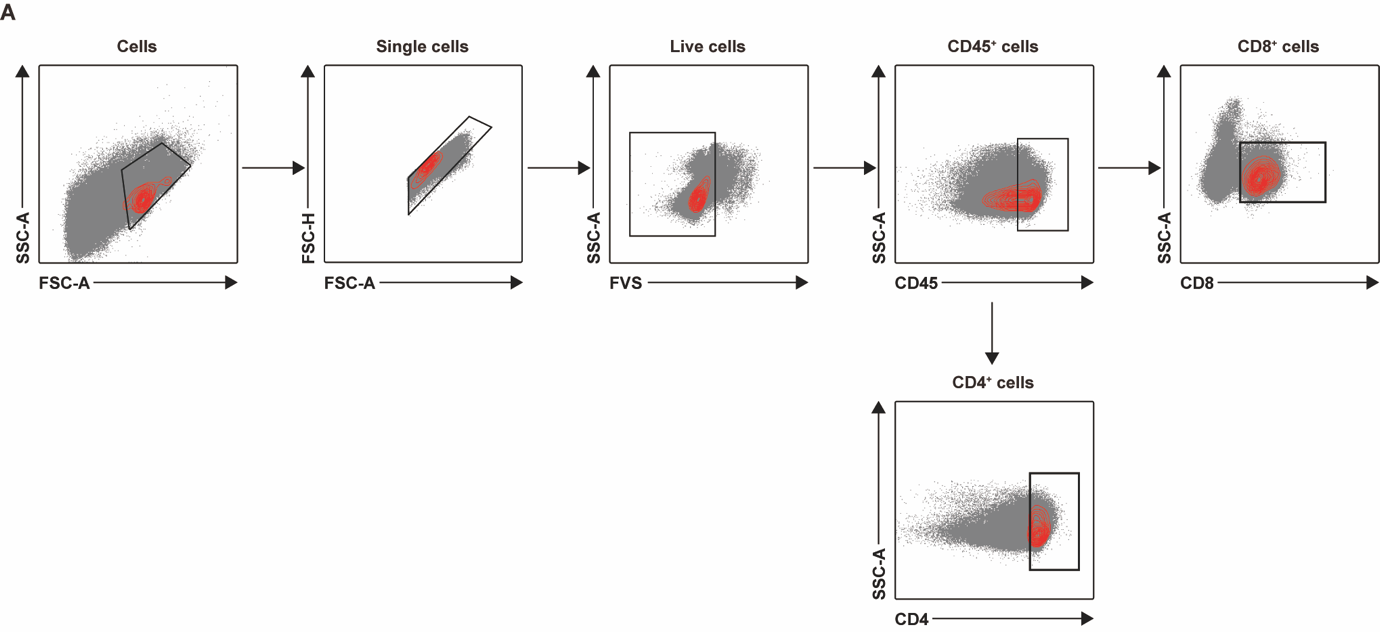


**Figure S5. Gating strategies used in Figure 3, 5, and 6.**

1. Gating strategies for CD4^+^ and CD8^+^ T cells in mice spleen (Related to Figure 3E, 5G-J, and 6D-I).


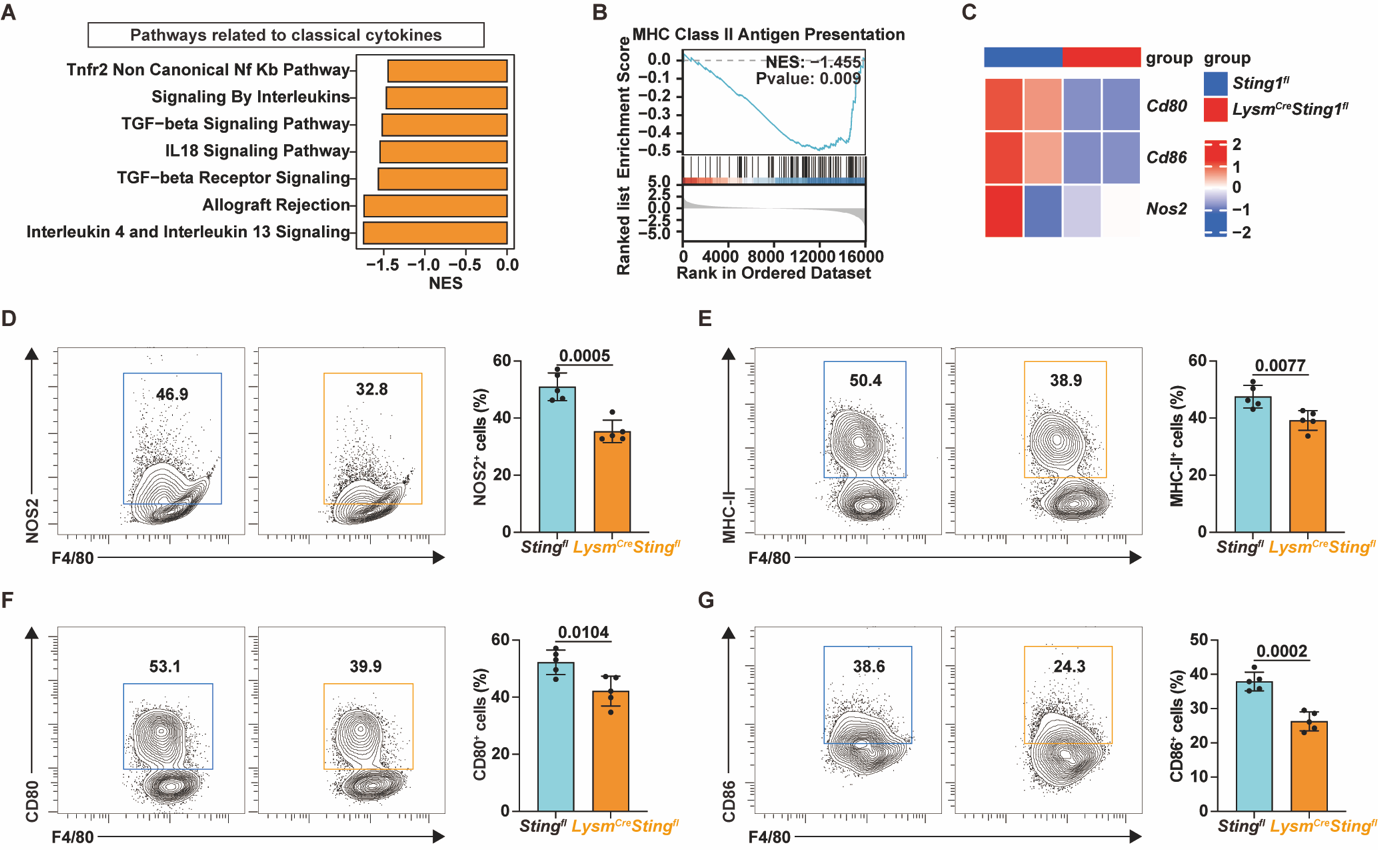


**Figure S6. *Sting* knockout inhibited antigen presenting function of macrophages**

1. Pathways related to classical cytokine signaling.
2. GSEA_KEGG showing the MHC Class II Antigen Presentation Pathway.
3. Heatmap displaying the genes encoding co-stimulatory molecules.

(D-G) Representative flow cytometry plots (left panel) and statistical charts (right panel) showing the proportion of (D) NOS2, (E) MHC-II, (F) CD80, or (G) CD86 expressing cells in splenic macrophages (n = 5 per group, mean ﻿± SD, Student’s *t* test).


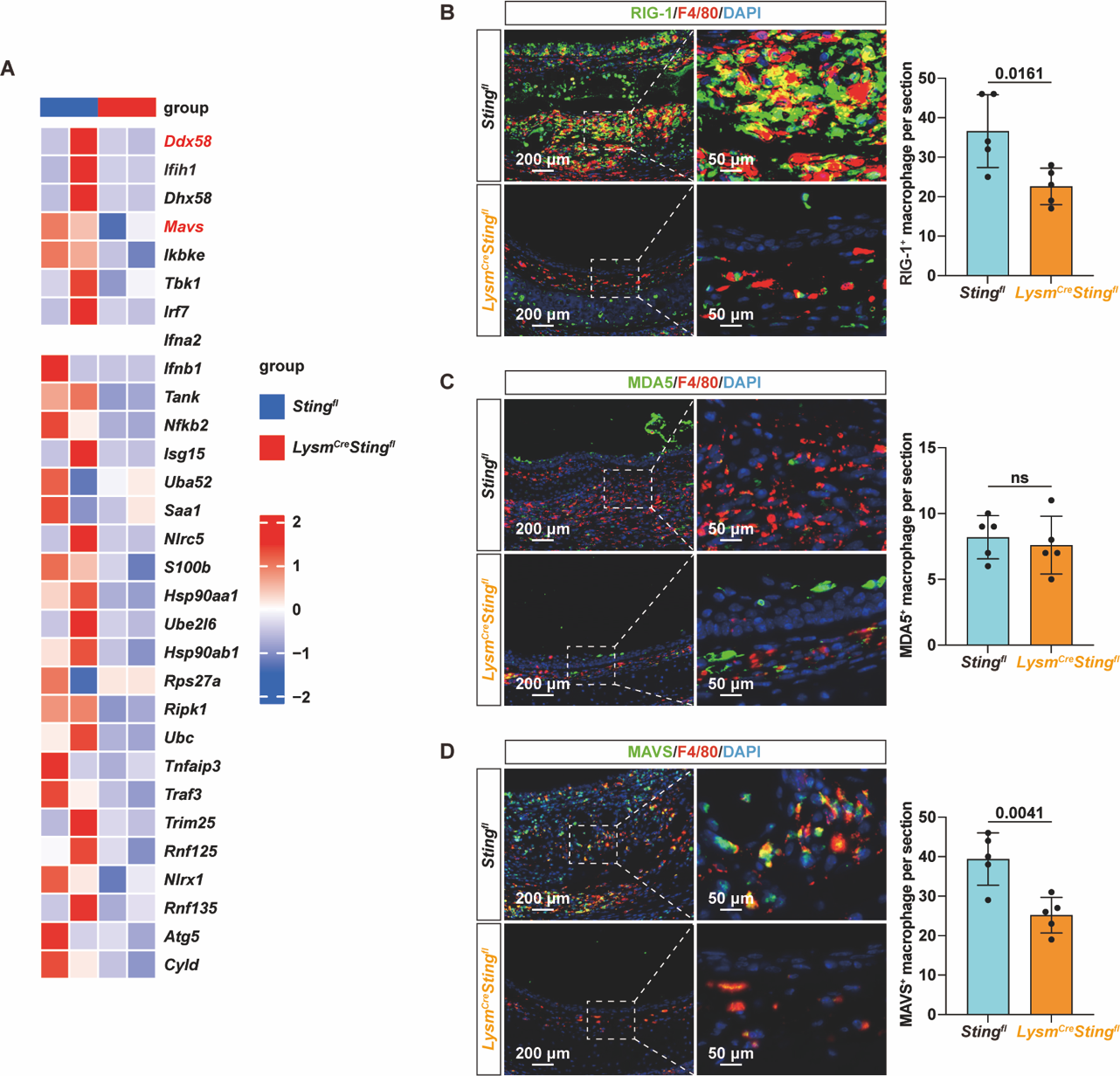


**Figure S7. Macrophage-specific *Sting1* deletion inhibited RIG-I/MAVS pathway in allografts.**

(A) Heatmap showing the genes enriched in the DDX58/IFIH1 Mediated Induction of Interferon Alpha/Beta pathway.

(B-D) Representative immunofluorescence images showing (B) RIG-1, (C) MDA5, and (D) MAVS in macrophages infiltrating allografts (n = 5 per group, mean ± SD, Student’s *t* test).


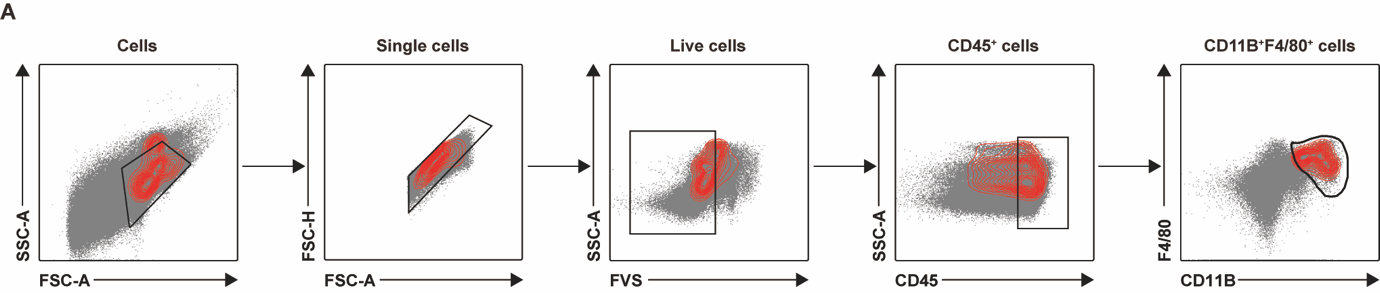


**Figure S8. Gating strategies used in Figure 4.**

1. Gating strategies for macrophages in mice spleen (Related to Figure 4H-I, and Figure S7D-G).


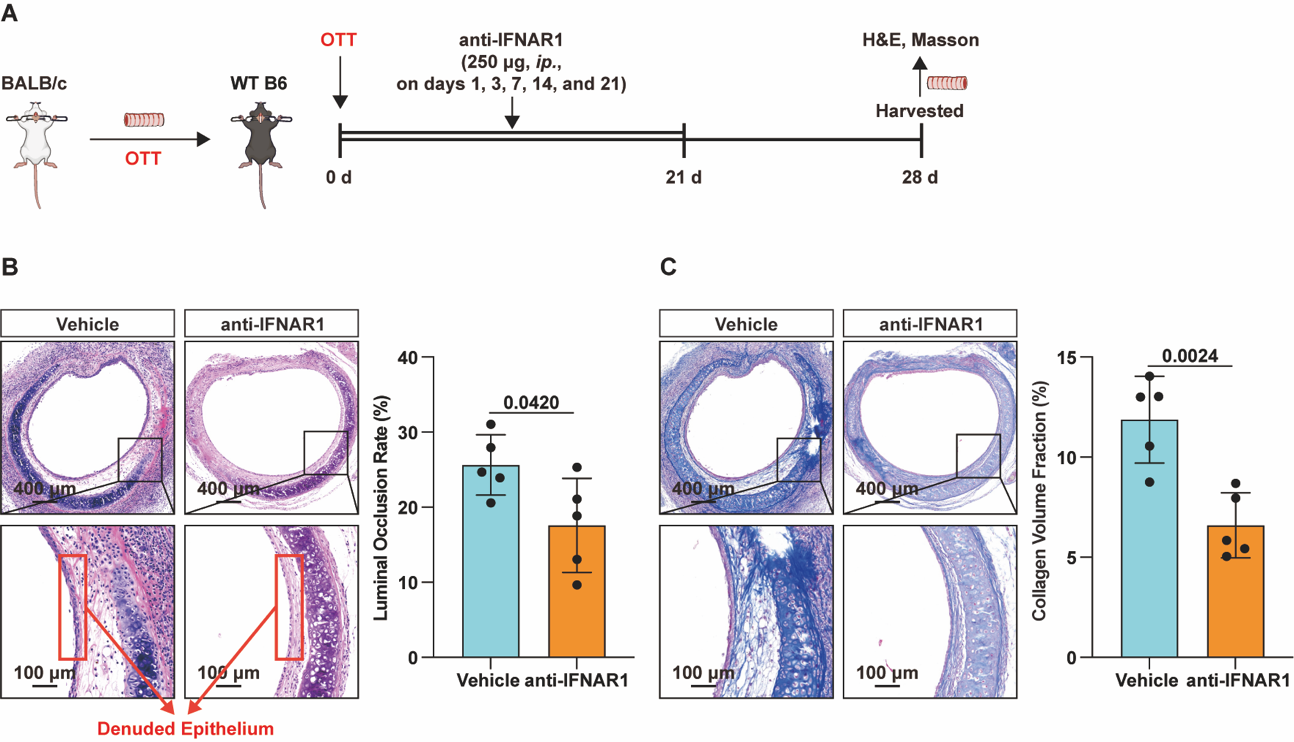


**Figure S9. Pharmacological inhibition of IFNAR alleviated OB**

(A) Experiment workflow: Recipient mice were intraperitoneally injected with anti-IFNAR1 to explore the role of the IFN-I and IFN-α in OB.

(B) Representative H&E-stained sections of trachea (left panel) and a statistical diagram (right panel) showing the degree of stenosis in tracheal allografts. (n = 5 per group, mean ± SD, Student’s *t* test).

(C) Representative Masson staining sections of trachea (left panel) and statistical charts (right panel) illustrating collagen deposition (n = 5 per group, mean ± SD, Student’s *t* test).

**Supplementary Materials and Methods**

**Orthotopic Tracheal Transplantation (OTT)**

The transplantation model was performed as previously described ^1,2^. Briefly, donor BALB/c mice were anesthetized via intraperitoneal injection of pentobarbital sodium. A tracheal segment consisting of six cartilage rings was aseptically harvested and rinsed with sterile saline. For the recipient B6 mice, the trachea was transected between the second and third cartilage rings below the cricoid cartilage. The donor tracheal graft was then orthotopically transplanted using end-to-end anastomosis with 10-0 sutures. Finally, the surgical incision was closed with 6-0 sutures.

**Animal Grouping**

Each mouse was assigned an identification number, which was used to generate random numbers for allocation into different groups. In the syngraft group, donor tracheas from B6 mice were transplanted into WT B6 recipient mice; in the allograft group, tracheas from BALB/c mice were transplanted into WT B6 recipient mice; in the *Sting1^fl^* or *Lysm^Cre^Sting1^fl^* groups, donor tracheas from BALB/c mice were transplanted into *Sting1^fl^* or *Lysm^Cre^Sting1^fl^* mice, respectively; in the RO8191, rIFN-α, and rIFN-β groups, tracheas from BALB/c mice were transplanted into *Lysm^Cre^Sting1^fl^* mice treated with RO8191, rIFN-α, and rIFN-β, respectively; in the CTLA4-Ig group, donor tracheas from BALB/c mice were transplanted into *Sting1^fl^* or *Lysm^Cre^Sting1^fl^* mice injected with CTLA4-Ig.

**Histology Assessment**

The H&E-stained sections were used to evaluate the luminal occlusion rate and the preservation of ciliated columnar epithelium, while Masson's trichrome staining was employed to detect subepithelial collagen deposition. The quantification methods were detailed in our previous publications ^1^. Briefly, the luminal occlusion rate was calculated as the ratio of occluded lumen area to total lumen area, and the collagen deposition rate was quantified as the ratio of subepithelial blue-stained area to total area.

**Immunofluorescence Staining**

Immunofluorescence staining was performed to detect infiltrating dsDNA^+^TOM20^+^F4/80⁺ cells, cGAS⁺F4/80⁺ cells, STING⁺F4/80⁺ cells, p-TBK1^+^F4/80⁺ cells, TBK1^+^F4/80⁺ cells, RIG-1^+^F4/80⁺ cells, MDA5^+^F4/80⁺ cells, MAVS^+^F4/80⁺ cells, TLR9^+^F4/80⁺ cells, Ly6G⁺ cells, CD4⁺ T cells, CD8⁺ T cells, as well as IFN-α and IFN-β in tracheal allografts collected on day 7 post-transplantation. The antibodies selected for immunofluorescence staining are listed in Table S1. The mean fluorescence intensity was measured by ImageJ software (version 1.54).

**Real-time Quantitative Polymerase Chain Reaction (RT-qPCR)**

RT-qPCR was performed to detect the mRNA levels of cytokines infiltrated in tracheal allografts. Briefly, total RNA was extracted from the allografts using TRIzol (Magen), followed by cDNA synthesis using ABScript II cDNA First-Strand Synthesis Kit (Cat# RK20400, ABclonal). Subsequently, qPCR was performed using ABScript III RT Master Mix for qPCR (Cat# RK20428, ABclonal). The relative mRNA levels were calculated using the 2^−ΔΔCT^ method. The primers used are shown in Table S1.

**Flow Cytometry**

Mouse spleens were mechanically dissociated, filtered, and subjected to red blood cell lysis before being resuspended as a single-cell suspension. Approximately 10⁷ cells from this suspension were sequentially stained with Fixable Viability Stain 510 (BD Biosciences, Cat# 564406), anti-CD16/32 antibodies (BD Biosciences, Cat# 553141), and fluorochrome-conjugated antibodies against surface antigens. Subsequently, the cells were permeabilized using the Transcription Factor Buffer Set (BD Biosciences, Cat# 562574), followed by intracellular staining. Antibodies used for flow cytometry are listed in Table S2. Data analysis was performed using FlowJo software (version 10.8.1).

**RNA sequencing and** **bioinformatics analysis**

For RNA sequencing of orthotopic tracheal grafts, we referenced the sampling protocol described in *Eur Respir J. 2021 Mar 11;57(3):2000344* (PMID: 33033147) ^3^. Specifically, grafts were harvested on day 7 after transplantation, when the allograft rejection peaked at this time point ^4^. And to meet the requirements of RNA sequencing, we extracted total RNA from four grafts and combined them into a single sample.

RNA sequencing was performed using the Illumina Novaseq 6000/MGISEQ-T7 sequencing platform at Shanghai Applied Protein Technology Co., Ltd. Differentially expressed genes (DEGs) were identified using DESeq2 (version 1.38) with criteria of fold change > 2.0 and adjusted P value < 0.05. Data processing and visualization were conducted using R software (version 4.2.2).

**Single-cell RNA sequencing and bioinformatics analysis**

The results in Figures 1E-H were derived from the re-analysis of a publicly available single-cell RNA sequencing dataset (GSE160760) published by Di Campli et al., which includes the sequencing of tracheal allografts and syngrafts in mice ^3^. Data analysis was performed using the Seurat R package (version 4.0.1). Cells with fewer than 500 genes, more than 5000 genes, or mitochondrial gene expression exceeding 20% were considered low quality and excluded. After data normalization using the "LogNormalize" function, identification of highly variable genes with the "FindVariableFeatures" function, data scaling with the "ScaleData" function, and clustering analysis using the "FindClusters" function, the "FindMarkers" function was employed to identify marker genes for each cell cluster. Based on these marker genes and existing biological knowledge, cell type annotations were assigned to each cell cluster.

**Enzyme-Linked Immunosorbent Assay (ELISA)**

Mouse plasma was collected for the detection of IFN-α and IFN-β. The experimental procedures adhered to the instructions provided with the assay kits. The ELISA kits utilized included the Mouse IFN-α ELISA Kit (Shanghai Jianglai Biotechnology Co., Ltd, Cat# JL12034) and the Mouse IFN-β ELISA Kit (Shanghai Jianglai Biotechnology Co., Ltd, Cat# JL20219).

**Supplementary Tables**

**Table S1. Key reagents table.**

| **Reagents** | **Source** | **Cat#** | **Url Link** |
| --- | --- | --- | --- |
| **Drugs and Cytokines** | | | |
| RO8191 | MedChemExpress | HY-W063968 | https://www.medchemexpress.cn/ro8191.html |
| Recombinant mouse IFN-α2 | R&D Systems | 10149-IF | https://www.bio-techne.com/p/proteins-enzymes/recombinant-mouse-ifn-alpha-2-ifna2-protein-cf_10149-if#tab-citations_reviews |
| Recombinant mouse IFN-β | Sigma | IF011 | https://www.sigmaaldrich.cn/CN/zh/product/mm/if011 |
| CTLA4-Ig | ﻿BioXCell | BE0099 | http://www.bioxcell.com.cn/in-vivo-antibodies/invivomab-recombinant-ctla-4-ig-hum-hum-be0099.html |
| **Antibodies for Immunofluorescence Staining** | | | |
| anti-cGAS  (1:500 dilution) | ProteinTech | 29958-1-AP | https://www.ptgcn.com/products/cGAS-Antibody-29958-1-AP.htm#publications |
| anti-F4/80  (1: 200) | Abcam | ab6640 | https://www.abcam.cn/products/primary-antibodies/f480-antibody-cia3-1-macrophage-marker-ab6640.html |
| anti-STING  (1: 200 dilution) | ProteinTech | 19851-1-AP | https://www.ptgcn.com/products/TMEM173-Antibody-19851-1-AP.htm |
| anti-RIG-1  (1: 200 dilution) | ProteinTech | 20566-1-AP | https://www.ptgcn.com/products/DDX58-Antibody-20566-1-AP.htm |
| anti-MDA5  (1: 200 dilution) | ABclonal | A13645 | https://abclonal.com.cn/catalog/A13645 |
| anti-MAVS  (1: 200 dilution) | ABclonal | A25005 | https://abclonal.com.cn/catalog/A25005 |
| anti-TLR9  (1: 200 dilution) | ABclonal | A14642 | https://abclonal.com.cn/catalog/A14642 |
| anti-dsDNA  (1: 200 dilution) | Abcam | Ab27156 | https://www.abcam.cn/products/primary-antibodies/ds-dna-antibody-35i9-dna-bsa-and-azide-free-ab27156.html |
| anti-p-TBK1  (1: 100 dilution) | Cell Signaling Technology | 5483S | https://www.cellsignal.cn/products/primary-antibodies/phospho-tbk1-nak-ser172-d52c2-xp-rabbit-mab/5483 |
| anti-TBK1 | Cell Signaling Technology | 38066S | https://www.cellsignal.cn/products/primary-antibodies/tbk1-nak-e8i3g-rabbit-mab/38066 |
| anti-Ly6G  (1: 400 dilution) | Invitrogen | MA1-82826 | https://www.thermofisher.cn/cn/zh/antibody/product/Ly-6G-Ly-6C-Antibody-clone-RB6-8C5-Monoclonal/MA1-82826 |
| anti-CD4  (1: 200 dilution) | ABclonal | A0363 | https://abclonal.com.cn/catalog/A0363 |
| anti-CD8  (1: 400 dilution) | ABclonal | A23305-PM | https://abclonal.com.cn/catalog/A23305-PM |
| anti-IFN-α  (1: 200 dilution) | ProteinTech | 18013-1-AP | https://www.ptgcn.com/products/IFNA1-Antibody-18013-1-AP.htm#product-information |
| anti-IFN-β  (1: 200 dilution) | ABclonal | A23651 | https://abclonal.com.cn/catalog/A23651 |
| **Primers** | | | |
| IFN-α | forward: 5'-TGCAGGAATTTCCCCTGACC-3'  reverse: 5'-AGACAGGGCTCTCCAGACTT-3' | | |
| IFN-β | forward: 5'-CCTGGAGCAGCTGAATGGAA-3'  reverse: 5'-CCACCCAGTGCT GGAGAAAT-3' | | |
| IL-1β | forward: 5'-TGCCACCTTTTGACAGTGAT-3'  reverse: 5'-GGAGCCTGTAGTGCAGTTGT-3' | | |
| CXCL10 | forward: 5'-AAGCTATGTGGAGGTGC GAC-3'  reverse: 5'-AACCCCTTGGGAAGATGGTG-3' | | |
| β-actin | forward: 5'-CGAGCACAGCTTCTTTGCAG-3'  reverse: 5'-CAGCACAGGGTGCTCCTCAG-3' | | |

**Table S2. Flow cytometry antibodies used in this study.**

| **Flow Cytometry Antibodies** | **Source** | **Cat#** | **Url Link** |
| --- | --- | --- | --- |
| FITC Conjugated Anti-Mouse IFN-Alpha | PBL Assay Science | 22100 | https://www.pblassaysci.com/antibodies/fitc-conjugated-anti-mouse-ifn-alpha-antibody-clone-rmma-1-mab-221003 |
| PE anti-mouse/human CD11b Antibody | Biolegend | 101207 | https://www.biolegend.com/en-us/products/pe-anti-mouse-human-cd11b-antibody-349 |
| Alexa Fluor® 594 anti-Nos2 (iNOS) Antibody | Biolegend | 696804 | https://www.biolegend.com/en-us/products/alexa-fluor-594-anti-nos2-antibody-15477 |
| PerCP anti-mouse F4/80 Antibody | Biolegend | 123126 | https://www.biolegend.com/en-us/products/percp-anti-mouse-f4-80-antibody-4302 |
| CD86 (B7-2) Monoclonal Antibody (GL1), PE-Cyanine7 | Invitrogen | 25-0862-82 | https://www.thermofisher.cn/cn/zh/antibody/product/CD86-B7-2-Antibody-clone-GL1-Monoclonal/25-0862-82 |
| IFN-β1 (D2J1D) Rabbit mAb | Cell Signaling Technology | 97450 | https://www.cellsignal.cn/products/primary-antibodies/ifn-b1-d2j1d-rabbit-mab/97450 |
| F(ab')2-Goat anti-Rabbit IgG (H+L) Cross-Adsorbed Secondary Antibody, APC | ﻿Invitrogen | 31984 | https://www.thermofisher.cn/cn/zh/antibody/product/Goat-anti-Rabbit-IgG-H-L-Cross-Adsorbed-Secondary-Antibody-Polyclonal/31984 |
| APC/Cyanine7 anti-mouse CD45 Antibody | Biolegend | 157204 | https://www.biolegend.com/en-us/products/apc-cyanine7-anti-mouse-cd45-antibody-19190 |
| Brilliant Violet 421™ anti-mouse CD80 Antibody | Biolegend | 104725 | https://www.biolegend.com/en-us/products/brilliant-violet-421-anti-mouse-cd80-antibody-7357 |
| MHC Class II (I-A/I-E) Monoclonal Antibody (M5/114.15.2), Brilliant Violet™ 650 | Invitrogen | 416-5321-82 | https://www.thermofisher.cn/cn/zh/antibody/product/MHC-Class-II-I-A-I-E-Antibody-clone-M5-114-15-2-Monoclonal/416-5321-82 |
| CD4 Monoclonal Antibody (GK1.5), FITC | ﻿Invitrogen | 11-0041-82 | <https://www.thermofisher.cn/cn/zh/antibody/product/CD4-Antibody-clone-GK1-5-Monoclonal/11-0041-82> |
| Perforin Monoclonal Antibody (eBioOMAK-D), PE | ﻿Invitrogen | 12-9392-82 | <https://www.thermofisher.cn/cn/zh/antibody/product/Perforin-Antibody-clone-eBioOMAK-D-Monoclonal/12-9392-82> |
| CD45 Monoclonal Antibody (30-F11), PE-Texas Red | ﻿Invitrogen | MCD4517 | https://www.thermofisher.cn/cn/zh/antibody/product/CD45-Antibody-clone-30-F11-Monoclonal/MCD4517 |
| IL-17A Monoclonal Antibody (eBio17B7), PerCP-Cyanine5.5 | ﻿Invitrogen | 45-7177-82 | https://www.thermofisher.cn/cn/zh/antibody/product/IL-17A-Antibody-clone-eBio17B7-Monoclonal/45-7177-82 |
| Granzyme B Monoclonal Antibody (NGZB), PE-Cyanine7 | ﻿Invitrogen | 25-8898-82 | https://www.thermofisher.cn/cn/zh/antibody/product/Granzyme-B-Antibody-clone-NGZB-Monoclonal/25-8898-82 |
| CD8a Monoclonal Antibody (53-6.7), APC | ﻿Invitrogen | 17-0081-82 | https://www.thermofisher.cn/cn/zh/antibody/product/CD8a-Antibody-clone-53-6-7-Monoclonal/17-0081-82 |
| FOXP3 Monoclonal Antibody (FJK-16s), Alexa Fluor™ 700 | ﻿Invitrogen | 56-5773-82 | https://www.thermofisher.cn/cn/zh/antibody/product/FOXP3-Antibody-clone-FJK-16s-Monoclonal/56-5773-82 |
| IFN gamma Monoclonal Antibody (XMG1.2), eFluor™ 450 | ﻿Invitrogen | 48-7311-82 | https://www.thermofisher.cn/cn/zh/antibody/product/IFN-gamma-Antibody-clone-XMG1-2-Monoclonal/48-7311-82 |
| CD44 Monoclonal Antibody (IM7), Brilliant Violet™ 605 | ﻿Invitrogen | 406-0441-82 | https://www.thermofisher.cn/cn/zh/antibody/product/CD44-Antibody-clone-IM7-Monoclonal/406-0441-82 |
| Ki-67 Monoclonal Antibody (SolA15), Brilliant Violet™ 650 | ﻿Invitrogen | 416-5698-82 | https://www.thermofisher.cn/cn/zh/antibody/product/Ki-67-Antibody-clone-SolA15-Monoclonal/416-5698-82 |

**Table S3. Statistical data in this study.**

| **Figure 1A** | | | | | | | | | | | | | | |
| --- | --- | --- | --- | --- | --- | --- | --- | --- | --- | --- | --- | --- | --- | --- |
| cGAS^+^ macrophages per section | | | | Syngraf | | | Allograft | | | | Student’s *t* test  *p*=0.0002 | | | |
|  |  |  |  | 10 | | | 31 | | | |  |  |  |  |
|  |  |  |  | 6 | | | 31 | | | |  |  |  |  |
|  |  |  |  | 14 | | | 34 | | | |  |  |  |  |
|  |  |  |  | 12 | | | 26 | | | |  |  |  |  |
|  |  |  |  | 7 | | | 20 | | | |  |  |  |  |
| **Figure 1B** | | | | | | | | | | | | | | |
| STING^+^ macrophages per section | | | | Syngraf | | | Allograft | | | | Student’s *t* test  *P*<0.0001 | | | |
|  |  |  |  | 12 | | | 37 | | | |  |  |  |  |
|  |  |  |  | 15 | | | 41 | | | |  |  |  |  |
|  |  |  |  | 23 | | | 49 | | | |  |  |  |  |
|  |  |  |  | 14 | | | 38 | | | |  |  |  |  |
|  |  |  |  | 14 | | | 43 | | | |  |  |  |  |
| **Figure 1C** | | | | | | | | | | | | | | |
| p-TBK1^+^ macrophages per section | | | | Syngraf | | | Allograft | | | | Student’s *t* test  *P*<0.0001 | | | |
|  |  |  |  | 9 | | | 43 | | | |  |  |  |  |
|  |  |  |  | 14 | | | 32 | | | |  |  |  |  |
|  |  |  |  | 17 | | | 28 | | | |  |  |  |  |
|  |  |  |  | 6 | | | 40 | | | |  |  |  |  |
|  |  |  |  | 13 | | | 37 | | | |  |  |  |  |
| **Figure 1D** | | | | | | | | | | | | | | |
| TBK1^+^ macrophages per section | | | | Syngraf | | | Allograft | | | | Student’s *t* test  *p*=0.0001 | | | |
|  |  |  |  | 10 | | | 20 | | | |  |  |  |  |
|  |  |  |  | 13 | | | 22 | | | |  |  |  |  |
|  |  |  |  | 8 | | | 22 | | | |  |  |  |  |
|  |  |  |  | 12 | | | 17 | | | |  |  |  |  |
|  |  |  |  | 10 | | | 18 | | | |  |  |  |  |
| **Figure 1E** | | | | | | | | | | | | | | |
| Fluorescence intensity of IFN-α | | | | Syngraf | | | Allograft | | | | Student’s *t* test  *p*=0.0002 | | | |
|  |  |  |  | 2.367 | | | 7.042 | | | |  |  |  |  |
|  |  |  |  | 3.652 | | | 5.671 | | | |  |  |  |  |
|  |  |  |  | 1.715 | | | 5.053 | | | |  |  |  |  |
|  |  |  |  | 1.452 | | | 6.873 | | | |  |  |  |  |
|  |  |  |  | 3.051 | | | 7.693 | | | |  |  |  |  |
| **Figure 1F** | | | | | | | | | | | | | | |
| Fluorescence intensity of IFN-β | | | | Syngraf | | | Allograft | | | | Student’s *t* test  *p*=0.0001 | | | |
|  |  |  |  | 1.929 | | | 8.394 | | | |  |  |  |  |
|  |  |  |  | 2.045 | | | 5.573 | | | |  |  |  |  |
|  |  |  |  | 3.105 | | | 7.062 | | | |  |  |  |  |
|  |  |  |  | 1.753 | | | 6.600 | | | |  |  |  |  |
|  |  |  |  | 1.204 | | | 5.184 | | | |  |  |  |  |
| **Figure 1G** | | | | | | | | | | | | | | |
| Relative mRNA expression of IFN-α | | | | Syngraf | | | Allograft | | | | Student’s *t* test  *p*=0.0005 | | | |
|  |  |  |  | 0.87998788 | | | 69.1770804 | | | |  |  |  |  |
|  |  |  |  | 0.93663286 | | | 54.7791720 | | | |  |  |  |  |
|  |  |  |  | 1.21326012 | | | 76.4029033 | | | |  |  |  |  |
| Relative mRNA expression of IFN-β | | | | Syngraf | | | Allograft | | | | Student’s *t* test  *p*=0.0004 | | | |
|  |  |  |  | 0.98699256 | | | 23.5520017 | | | |  |  |  |  |
|  |  |  |  | 0.98017490 | | | 17.5627206 | | | |  |  |  |  |
|  |  |  |  | 1.03367150 | | | 22.6448505 | | | |  |  |  |  |
| Relative mRNA expression of IL-1β | | | | Syngraf | | | Allograft | | | | Student’s *t* test  *p*=0.0001 | | | |
|  |  |  |  | 1.12246205 | | | 64.2964272 | | | |  |  |  |  |
|  |  |  |  | 0.95263800 | | | 63.1188931 | | | |  |  |  |  |
|  |  |  |  | 0.93519125 | | | 77.1717010 | | | |  |  |  |  |
| Relative mRNA expression of IL-1β | | | | Syngraf | | | Allograft | | | | Student’s *t* test  *p*=0.0013 | | | |
|  |  |  |  | 0.75146498 | | | 46.1346171 | | | |  |  |  |  |
|  |  |  |  | 0.91031922 | | | 60.0369082 | | | |  |  |  |  |
|  |  |  |  | 1.46183226 | | | 71.2315483 | | | |  |  |  |  |
| **Figure 2B** | | | | | | | | | | | | | | |
| Luminal Occlusion Rate | | | | *Sting^fl^* | | | *Lysm^Cre^Sting^fl^* | | | | Student’s *t* test  *p*=0.0012 | | | |
|  |  |  |  | 30.24 | | | 11.60 | | | |  |  |  |  |
|  |  |  |  | 23.91 | | | 14.30 | | | |  |  |  |  |
|  |  |  |  | 24.80 | | | 17.04 | | | |  |  |  |  |
|  |  |  |  | 31.04 | | | 16.46 | | | |  |  |  |  |
|  |  |  |  | 21.64 | | | 18.54 | | | |  |  |  |  |
| **Figure 2C** | | | | | | | | | | | | | | |
| Collagen Volume Fraction | | | | *Sting^fl^* | | | *Lysm^Cre^Sting^fl^* | | | | Student’s *t* test  *p*=0.0096 | | | |
|  |  |  |  | 14.04 | | | 6.04 | | | |  |  |  |  |
|  |  |  |  | 12.17 | | | 4.27 | | | |  |  |  |  |
|  |  |  |  | 10.46 | | | 6.75 | | | |  |  |  |  |
|  |  |  |  | 7.05 | | | 5.05 | | | |  |  |  |  |
|  |  |  |  | 8.93 | | | 7.62 | | | |  |  |  |  |
| **Figure 2D** | | | | | | | | | | | | | | |
| Average optical density of IL-1β | | | | *Sting^fl^* | | | *Lysm^Cre^Sting^fl^* | | | | Student’s *t* test  *p*=0.0008 | | | |
|  |  |  |  | 24.6 | | | 11.3 | | | |  |  |  |  |
|  |  |  |  | 27.3 | | | 14.6 | | | |  |  |  |  |
|  |  |  |  | 19.1 | | | 16.9 | | | |  |  |  |  |
|  |  |  |  | 22.8 | | | 7.4 | | | |  |  |  |  |
|  |  |  |  | 28.0 | | | 9.4 | | | |  |  |  |  |
| **Figure 2E** | | | | | | | | | | | | | | |
| Average optical density of TNF-α | | | | *Sting^fl^* | | | *Lysm^Cre^Sting^fl^* | | | | Student’s *t* test  *p*=0.0148 | | | |
|  |  |  |  | 20.3 | | | 9.4 | | | |  |  |  |  |
|  |  |  |  | 14.2 | | | 14.8 | | | |  |  |  |  |
|  |  |  |  | 16.2 | | | 13.9 | | | |  |  |  |  |
|  |  |  |  | 22.7 | | | 11.0 | | | |  |  |  |  |
|  |  |  |  | 15.9 | | | 11.5 | | | |  |  |  |  |
| **Figure 3A** | | | | | | | | | | | | | | |
| Fluorescence intensity of Ly6G | | | | *Sting^fl^* | | | *Lysm^Cre^Sting^fl^* | | | | Student’s *t* test  *p*=0.0004 | | | |
|  |  |  |  | 3.416 | | | 1.402 | | | |  |  |  |  |
|  |  |  |  | 3.197 | | | 1.622 | | | |  |  |  |  |
|  |  |  |  | 3.094 | | | 1.852 | | | |  |  |  |  |
|  |  |  |  | 2.703 | | | 2.304 | | | |  |  |  |  |
|  |  |  |  | 2.785 | | | 2.04 | | | |  |  |  |  |
| **Figure 3B** | | | | | | | | | | | | | | |
| Fluorescence intensity of F4/80 | | | | *Sting^fl^* | | | *Lysm^Cre^Sting^fl^* | | | | Student’s *t* test  *p*=0.0019 | | | |
|  |  |  |  | 4.033 | | | 1.788 | | | |  |  |  |  |
|  |  |  |  | 4.204 | | | 2.045 | | | |  |  |  |  |
|  |  |  |  | 3.463 | | | 2.464 | | | |  |  |  |  |
|  |  |  |  | 3.010 | | | 2.584 | | | |  |  |  |  |
|  |  |  |  | 3.868 | | | 2.956 | | | |  |  |  |  |
| **Figure 3C** | | | | | | | | | | | | | | |
| Fluorescence intensity of CD4 | | | | *Sting^fl^* | | | *Lysm^Cre^Sting^fl^* | | | | Student’s *t* test  *p*=0.0010 | | | |
|  |  |  |  | 2.742 | | | 1.557 | | | |  |  |  |  |
|  |  |  |  | 3.042 | | | 1.645 | | | |  |  |  |  |
|  |  |  |  | 2.481 | | | 1.950 | | | |  |  |  |  |
|  |  |  |  | 2.204 | | | 2.053 | | | |  |  |  |  |
|  |  |  |  | 2.658 | | | 1.654 | | | |  |  |  |  |
| **Figure 3D** | | | | | | | | | | | | | | |
| Fluorescence intensity of CD8 | | | | *Sting^fl^* | | | *Lysm^Cre^Sting^fl^* | | | | Student’s *t* test  *p*=0.0011 | | | |
|  |  |  |  | 2.229 | | | 1.389 | | | |  |  |  |  |
|  |  |  |  | 2.547 | | | 1.492 | | | |  |  |  |  |
|  |  |  |  | 2.042 | | | 1.689 | | | |  |  |  |  |
|  |  |  |  | 1.894 | | | 1.704 | | | |  |  |  |  |
|  |  |  |  | 2.484 | | | 1.396 | | | |  |  |  |  |
| **Figure 3E** | | | | | | | | | | | | | | |
| CD44^+^ populations in CD8^+^ T cells | | | | *Sting^fl^* | | | *Lysm^Cre^Sting^fl^* | | | | Student’s *t* test  *p*=0.0003 | | | |
|  |  |  |  | 31.2 | | | 16.2 | | | |  |  |  |  |
|  |  |  |  | 35.6 | | | 17.3 | | | |  |  |  |  |
|  |  |  |  | 26.6 | | | 19.4 | | | |  |  |  |  |
|  |  |  |  | 24.6 | | | 16.7 | | | |  |  |  |  |
|  |  |  |  | 32.2 | | | 16.6 | | | |  |  |  |  |
| Ki-67^+^ populations in CD8^+^ T cells | | | | *Sting^fl^* | | | *Lysm^Cre^Sting^fl^* | | | | Student’s *t* test  *p*=0.0007 | | | |
|  |  |  |  | 31.7 | | | 17.2 | | | |  |  |  |  |
|  |  |  |  | 35.9 | | | 20.5 | | | |  |  |  |  |
|  |  |  |  | 30.4 | | | 18.5 | | | |  |  |  |  |
|  |  |  |  | 24.6 | | | 17.9 | | | |  |  |  |  |
|  |  |  |  | 28.5 | | | 22.2 | | | |  |  |  |  |
| IFN-γ^+^ populations in CD8^+^ T cells | | | | *Sting^fl^* | | | *Lysm^Cre^Sting^fl^* | | | | Student’s *t* test  *p*=0.0020 | | | |
|  |  |  |  | 18.3 | | | 7.22 | | | |  |  |  |  |
|  |  |  |  | 14.8 | | | 12.80 | | | |  |  |  |  |
|  |  |  |  | 17.5 | | | 5.30 | | | |  |  |  |  |
|  |  |  |  | 14.8 | | | 10.80 | | | |  |  |  |  |
|  |  |  |  | 19.9 | | | 11.0 | | | |  |  |  |  |
| Perforin^+^ populations in CD8^+^ T cells | | | | *Sting^fl^* | | | *Lysm^Cre^Sting^fl^* | | | | Student’s *t* test  *p*=0.0031 | | | |
|  |  |  |  | 12.02 | | | 8.13 | | | |  |  |  |  |
|  |  |  |  | 14.28 | | | 9.75 | | | |  |  |  |  |
|  |  |  |  | 11.40 | | | 6.01 | | | |  |  |  |  |
|  |  |  |  | 10.50 | | | 7.46 | | | |  |  |  |  |
|  |  |  |  | 10.41 | | | 7.90 | | | |  |  |  |  |
| GZMB^+^ populations in CD8^+^ T cells | | | | *Sting^fl^* | | | *Lysm^Cre^Sting^fl^* | | | | Student’s *t* test  *p*=0.0001 | | | |
|  |  |  |  | 9.07 | | | 2.28 | | | |  |  |  |  |
|  |  |  |  | 7.07 | | | 4.06 | | | |  |  |  |  |
|  |  |  |  | 7.45 | | | 1.98 | | | |  |  |  |  |
|  |  |  |  | 6.83 | | | 2.85 | | | |  |  |  |  |
|  |  |  |  | 10.05 | | | 3.56 | | | |  |  |  |  |
| CD44^+^ populations in CD4^+^ T cells | | | | *Sting^fl^* | | | *Lysm^Cre^Sting^fl^* | | | | Student’s *t* test  *p*=0.0003 | | | |
|  |  |  |  | 23.7 | | | 13.2 | | | |  |  |  |  |
|  |  |  |  | 27.6 | | | 16.4 | | | |  |  |  |  |
|  |  |  |  | 26.4 | | | 14.0 | | | |  |  |  |  |
|  |  |  |  | 33.0 | | | 15.6 | | | |  |  |  |  |
|  |  |  |  | 29.5 | | | 20.5 | | | |  |  |  |  |
| Ki-67^+^ populations in CD4^+^ T cells | | | | *Sting^fl^* | | | *Lysm^Cre^Sting^fl^* | | | | Student’s *t* test  *p*=0.0019 | | | |
|  |  |  |  | 21.1 | | | 11.3 | | | |  |  |  |  |
|  |  |  |  | 22.5 | | | 14.0 | | | |  |  |  |  |
|  |  |  |  | 18.4 | | | 8.40 | | | |  |  |  |  |
|  |  |  |  | 27.5 | | | 9.50 | | | |  |  |  |  |
|  |  |  |  | 16.5 | | | 13.50 | | | |  |  |  |  |
| FOXP3^+^ populations in CD4^+^ T cells | | | | *Sting^fl^* | | | *Lysm^Cre^Sting^fl^* | | | | Student’s *t* test  *p*=0.0064 | | | |
|  |  |  |  | 13.0 | | | 21.0 | | | |  |  |  |  |
|  |  |  |  | 13.8 | | | 19.4 | | | |  |  |  |  |
|  |  |  |  | 14.3 | | | 16.3 | | | |  |  |  |  |
|  |  |  |  | 15.4 | | | 15.9 | | | |  |  |  |  |
|  |  |  |  | 15.3 | | | 18.6 | | | |  |  |  |  |
| IFN-γ^+^ populations in CD4^+^ T cells | | | | *Sting^fl^* | | | *Lysm^Cre^Sting^fl^* | | | | Student’s *t* test  *p*>0.05 | | | |
|  |  |  |  | 31.07 | | | 29.89 | | | |  |  |  |  |
|  |  |  |  | 28.02 | | | 22.42 | | | |  |  |  |  |
|  |  |  |  | 27.47 | | | 26.95 | | | |  |  |  |  |
|  |  |  |  | 23.04 | | | 23.04 | | | |  |  |  |  |
|  |  |  |  | 21.42 | | | 19.81 | | | |  |  |  |  |
| IL-17A^+^ populations in CD4^+^ T cells | | | | *Sting^fl^* | | | *Lysm^Cre^Sting^fl^* | | | | Student’s *t* test  *p*>0.05 | | | |
|  |  |  |  | 13.95 | | | 13.77 | | | |  |  |  |  |
|  |  |  |  | 12.04 | | | 11.76 | | | |  |  |  |  |
|  |  |  |  | 9.79 | | | 8.51 | | | |  |  |  |  |
|  |  |  |  | 8.67 | | | 8.40 | | | |  |  |  |  |
|  |  |  |  | 10.68 | | | 9.67 | | | |  |  |  |  |
| **Figure 4D** | | | | | | | | | | | | | | |
| Fluorescence intensity of IFN-α | | | | *Sting^fl^* | | | *Lysm^Cre^Sting^fl^* | | | | Student’s *t* test  *p*=0.0001 | | | |
|  |  |  |  | 8.065 | | | 2.373 | | | |  |  |  |  |
|  |  |  |  | 8.042 | | | 2.684 | | | |  |  |  |  |
|  |  |  |  | 7.284 | | | 3.045 | | | |  |  |  |  |
|  |  |  |  | 5.446 | | | 3.742 | | | |  |  |  |  |
|  |  |  |  | 6.472 | | | 3.624 | | | |  |  |  |  |
| **Figure 4E** | | | | | | | | | | | | | | |
| Fluorescence intensity of IFN-β | | | | *Sting^fl^* | | | *Lysm^Cre^Sting^fl^* | | | | Student’s *t* test  *p*=0.0012 | | | |
|  |  |  |  | 10.863 | | | 1.422 | | | |  |  |  |  |
|  |  |  |  | 7.042 | | | 3.024 | | | |  |  |  |  |
|  |  |  |  | 6.426 | | | 1.977 | | | |  |  |  |  |
|  |  |  |  | 8.472 | | | 3.674 | | | |  |  |  |  |
|  |  |  |  | 5.634 | | | 3.420 | | | |  |  |  |  |
| **Figure 4F** | | | | | | | | | | | | | | |
| Relative expression of IFN-α mRNA | | | | *Sting^fl^* | | | *Lysm^Cre^Sting^fl^* | | | | Student’s *t* test  *P*<0.0001 | | | |
|  |  |  |  | 38.26033970 | | | 1.25798185 | | | |  |  |  |  |
|  |  |  |  | 35.12544130 | | | 0.79614941 | | | |  |  |  |  |
|  |  |  |  | 41.57884500 | | | 0.99846086 | | | |  |  |  |  |
| Relative expression of IFN-β mRNA | | | | *Sting^fl^* | | | *Lysm^Cre^Sting^fl^* | | | | Student’s *t* test  *P*=0.0001 | | | |
|  |  |  |  | 15.11362450 | | | 0.87525650 | | | |  |  |  |  |
|  |  |  |  | 13.93954060 | | | 1.02890595 | | | |  |  |  |  |
|  |  |  |  | 17.32092950 | | | 1.11042435 | | | |  |  |  |  |
| **Figure 4G** | | | | | | | | | | | | | | |
| IFN-α in plasma | | | | *Sting^fl^* | | | *Lysm^Cre^Sting^fl^* | | | | Student’s *t* test  *P*<0.0001 | | | |
|  |  |  |  | 472.10 | | | 139.21 | | | |  |  |  |  |
|  |  |  |  | 446.14 | | | 158.04 | | | |  |  |  |  |
|  |  |  |  | 507.09 | | | 97.27 | | | |  |  |  |  |
|  |  |  |  | 455.01 | | | 169.05 | | | |  |  |  |  |
|  |  |  |  | 421.83 | | | 114.28 | | | |  |  |  |  |
| IFN-β in plasma | | | | *Sting^fl^* | | | *Lysm^Cre^Sting^fl^* | | | | Student’s *t* test  *P*<0.0001 | | | |
|  |  |  |  | 207.42 | | | 95.04 | | | |  |  |  |  |
|  |  |  |  | 201.40 | | | 85.42 | | | |  |  |  |  |
|  |  |  |  | 178.04 | | | 72.01 | | | |  |  |  |  |
|  |  |  |  | 246.53 | | | 103.74 | | | |  |  |  |  |
|  |  |  |  | 231.05 | | | 66.00 | | | |  |  |  |  |
| **Figure 4H** | | | | | | | | | | | | | | |
| IFN-α^+^ cells in macrophages | | | | *Sting^fl^* | | | *Lysm^Cre^Sting^fl^* | | | | Student’s *t* test  *P*=0.0006 | | | |
|  |  |  |  | 35.3 | | | 26.2 | | | |  |  |  |  |
|  |  |  |  | 37.1 | | | 22.5 | | | |  |  |  |  |
|  |  |  |  | 40.6 | | | 27.3 | | | |  |  |  |  |
|  |  |  |  | 31.0 | | | 28.0 | | | |  |  |  |  |
|  |  |  |  | 40.0 | | | 24.8 | | | |  |  |  |  |
| **Figure 4I** | | | | | | | | | | | | | | |
| IFN-β^+^ cells in macrophages | | | | *Sting^fl^* | | | *Lysm^Cre^Sting^fl^* | | | | Student’s *t* test  *P*=0.0033 | | | |
|  |  |  |  | 27.5 | | | 17.9 | | | |  |  |  |  |
|  |  |  |  | 31.2 | | | 22.1 | | | |  |  |  |  |
|  |  |  |  | 24.8 | | | 16.3 | | | |  |  |  |  |
|  |  |  |  | 26.3 | | | 19.6 | | | |  |  |  |  |
|  |  |  |  | 22.4 | | | 20.0 | | | |  |  |  |  |
| **Figure 5A** | | | | | | | | | | | | | | |
| Luminal Occlusion Rate | PBS | | RO8191 | | | rIFN-α | | 500 IU  rIFN-β | 1000 IU  rIFN-β | | | 1500 IU  rIFN-β | | Two-way ANOVA test |
|  | 10.50 | | 27.57 | | | 30.04 | | 15.05 | 16.46 | | | 16.47 | |  |
|  | 13.71 | | 24.05 | | | 22.57 | | 12.75 | 14.05 | | | 14.04 | |  |
|  | 16.40 | | 25.62 | | | 19.47 | | 16.97 | 11.57 | | | 12.04 | |  |
|  | 16.79 | | 21.04 | | | 23.05 | | 12.85 | 18.05 | | | 15.56 | |  |
|  | 19.02 | | 22.33 | | | 26.67 | | 11.50 | 15.75 | | | 15.84 | |  |
| **Figure 5B** | | | | | | | | | | | | | | |
| Collagen Volume Fraction | PBS | | RO8191 | | | rIFN-α | | 500 IU  rIFN-β | 1000 IU  rIFN-β | | | 1500 IU  rIFN-β | | Two-way ANOVA test |
|  | 7.03 | | 11.04 | | | 12.56 | | 8.04 | 8.04 | | | 7.44 | |  |
|  | 5.64 | | 10.46 | | | 11.05 | | 4.57 | 7.46 | | | 6.04 | |  |
|  | 9.04 | | 10.06 | | | 9.56 | | 5.00 | 6.58 | | | 5.59 | |  |
|  | 6.47 | | 9.07 | | | 12.55 | | 7.47 | 5.56 | | | 7.74 | |  |
|  | 7.54 | | 10.67 | | | 13.85 | | 5.42 | 4.05 | | | 6.05 | |  |
| **Figure 5C** | | | | | | | | | | | | | | |
| Fluorescence intensity of Ly6G | PBS | | RO8191 | | | rIFN-α | | 500 IU  rIFN-β | 1000 IU  rIFN-β | | | 1500 IU  rIFN-β | | Two-way ANOVA test |
|  | 1.837 | | 1.916 | | | 3.022 | | 2.016 | 2.134 | | | 2.108 | |  |
|  | 1.804 | | 2.129 | | | 3.156 | | 2.205 | 1.842 | | | 2.204 | |  |
|  | 1.627 | | 2.011 | | | 3.442 | | 1.742 | 2.402 | | | 2.042 | |  |
|  | 2.239 | | 2.548 | | | 2.302 | | 1.946 | 1.748 | | | 1.848 | |  |
|  | 2.102 | | 1.711 | | | 2.741 | | 2.418 | 1.904 | | | 2.104 | |  |
| **Figure 5D** | | | | | | | | | | | | | | |
| Fluorescence intensity of Ly6G | PBS | | RO8191 | | | rIFN-α | | 500 IU  rIFN-β | 1000 IU  rIFN-β | | | 1500 IU  rIFN-β | | Two-way ANOVA test |
|  | 2.014 | | 4.247 | | | 4.014 | | 2.305 | 2.716 | | | 2.044 | |  |
|  | 2.425 | | 4.174 | | | 3.890 | | 2.042 | 3.219 | | | 2.452 | |  |
|  | 2.705 | | 3.402 | | | 4.604 | | 2.394 | 3.414 | | | 2.195 | |  |
|  | 1.806 | | 3.801 | | | 3.664 | | 2.105 | 3.014 | | | 1.952 | |  |
|  | 2.205 | | 3.022 | | | 3.202 | | 2.492 | 2.500 | | | 2.504 | |  |
| **Figure 5E** | | | | | | | | | | | | | | |
| Fluorescence intensity of Ly6G | PBS | | RO8191 | | | rIFN-α | | 500 IU  rIFN-β | 1000 IU  rIFN-β | | | 1500 IU  rIFN-β | | Two-way ANOVA test |
|  | 1.705 | | 3.442 | | | 2.742 | | 2.041 | 2.104 | | | 2.174 | |  |
|  | 1.840 | | 2.890 | | | 2.649 | | 1.740 | 1.906 | | | 2.395 | |  |
|  | 2.104 | | 3.519 | | | 2.501 | | 2.404 | 2.104 | | | 2.205 | |  |
|  | 1.584 | | 2.640 | | | 3.194 | | 2.100 | 2.405 | | | 1.950 | |  |
|  | 2.094 | | 2.103 | | | 2.590 | | 1.850 | 2.275 | | | 1.983 | |  |
| **Figure 5F** | | | | | | | | | | | | | | |
| Fluorescence intensity of Ly6G | PBS | | RO8191 | | | rIFN-α | | 500 IU  rIFN-β | 1000 IU  rIFN-β | | | 1500 IU  rIFN-β | | Two-way ANOVA test |
|  | 1.649 | | 2.592 | | | 2.606 | | 1.584 | 1.706 | | | 1.793 | |  |
|  | 1.704 | | 2.214 | | | 2.993 | | 1.705 | 1.905 | | | 1.952 | |  |
|  | 2.051 | | 2.704 | | | 2.395 | | 1.953 | 2.195 | | | 2.042 | |  |
|  | 1.806 | | 2.5 | | | 2.862 | | 1.854 | 2.15 | | | 2.044 | |  |
|  | 2.257 | | 2.34 | | | 2.411 | | 1.807 | 1.953 | | | 2.375 | |  |
| **Figure 5G** | | | | | | | | | | | | | | |
| Fluorescence intensity of Ly6G | PBS | | RO8191 | | | rIFN-α | | 500 IU  rIFN-β | 1000 IU  rIFN-β | | | 1500 IU  rIFN-β | | Two-way ANOVA test |
|  | 19.5 | | 26.3 | | | 26.9 | | 20.1 | 22.5 | | | 23.6 | |  |
|  | 21.4 | | 26.5 | | | 27.4 | | 22.5 | 21.4 | | | 23.6 | |  |
|  | 17.3 | | 24.6 | | | 23.7 | | 23.6 | 19.5 | | | 20.5 | |  |
|  | 22.5 | | 23.3 | | | 28.5 | | 18.9 | 23.6 | | | 24.4 | |  |
|  | 18.6 | | 23.4 | | | 23.0 | | 20.4 | 20.5 | | | 21.6 | |  |
| **Figure 5H** | | | | | | | | | | | | | | |
| Fluorescence intensity of Ly6G | PBS | | RO8191 | | | rIFN-α | | 500 IU  rIFN-β | 1000 IU  rIFN-β | | | 1500 IU  rIFN-β | | Two-way ANOVA test |
|  | 7.160 | | 16.340 | | | 14.050 | | 7.480 | 8.140 | | | 8.040 | |  |
|  | 8.190 | | 15.050 | | | 11.530 | | 8.620 | 8.805 | | | 9.174 | |  |
|  | 6.020 | | 13.950 | | | 16.060 | | 6.500 | 9.175 | | | 9.846 | |  |
|  | 9.170 | | 17.600 | | | 10.180 | | 6.290 | 9.148 | | | 7.051 | |  |
|  | 5.470 | | 12.500 | | | 10.060 | | 8.950 | 6.830 | | | 6.175 | |  |
| **Figure 5I** | | | | | | | | | | | | | | |
| Fluorescence intensity of Ly6G | PBS | | RO8191 | | | rIFN-α | | 500 IU  rIFN-β | 1000 IU  rIFN-β | | | 1500 IU  rIFN-β | | Two-way ANOVA test |
|  | 11.0 | | 15.4 | | | 16.2 | | 12.0 | 11.2 | | | 11.9 | |  |
|  | 10.3 | | 16.8 | | | 17.8 | | 11.4 | 12.6 | | | 13.0 | |  |
|  | 9.8 | | 13.0 | | | 13.5 | | 13.0 | 11.9 | | | 14.0 | |  |
|  | 8.5 | | 13.7 | | | 15.2 | | 12.5 | 10.3 | | | 13.4 | |  |
|  | 12.7 | | 15.2 | | | 14.1 | | 9.8 | 9.2 | | | 14.6 | |  |
| **Figure 5J** | | | | | | | | | | | | | | |
| Fluorescence intensity of Ly6G | PBS | | RO8191 | | | rIFN-α | | 500 IU  rIFN-β | 1000 IU  rIFN-β | | | 1500 IU  rIFN-β | | Two-way ANOVA test |
|  | 16.5 | | 14.5 | | | 13.2 | | 14.4 | 16.8 | | | 16.8 | |  |
|  | 17.9 | | 13.6 | | | 13.9 | | 14.4 | 15.4 | | | 15.4 | |  |
|  | 17.4 | | 15.6 | | | 14.6 | | 13.9 | 17.2 | | | 17.2 | |  |
|  | 18.3 | | 12.9 | | | 16.2 | | 13.4 | 16.5 | | | 16.5 | |  |
|  | 19.0 | | 15.1 | | | 15.1 | | 14.7 | 15.6 | | | 15.6 | |  |
| **Figure 6B** | | | | | | | | | | | | | | |
| Luminal Occlusion Rate | | *Sting^fl^*  +Vehicle | | | *Sting^fl^*  +CTLA4-Ig | | | *Lysm^Cre^Sting^fl^* + Vehicle | | *Lysm^Cre^Sting^fl^* + CTLA4-Ig | | | Two-way ANOVA test | |
|  |  | 28.18 | | | 12.57 | | | 14.19 | | 14.04 | | |  |  |
|  |  | 25.96 | | | 14.59 | | | 16.95 | | 12.47 | | |  |  |
|  |  | 23.20 | | | 18.95 | | | 17.98 | | 17.58 | | |  |  |
|  |  | 31.18 | | | 17.4 | | | 16.81 | | 18.29 | | |  |  |
|  |  | 21.05 | | | 16.05 | | | 19.68 | | 15.06 | | |  |  |
| **Figure 6C** | | | | | | | | | | | | | | |
| Collagen Volume Fraction | | *Sting^fl^*  +Vehicle | | | *Sting^fl^*  +CTLA4-Ig | | | *Lysm^Cre^Sting^fl^* + Vehicle | | *Lysm^Cre^Sting^fl^* + CTLA4-Ig | | | Two-way ANOVA test | |
|  |  | 13.89 | | | 8.10 | | | 7.04 | | 4.00 | | |  |  |
|  |  | 11.95 | | | 6.67 | | | 4.19 | | 3.28 | | |  |  |
|  |  | 11.02 | | | 4.96 | | | 5.90 | | 5.04 | | |  |  |
|  |  | 7.57 | | | 5.77 | | | 5.79 | | 3.68 | | |  |  |
|  |  | 9.17 | | | 6.68 | | | 7.32 | | 2.68 | | |  |  |
| **Figure 6D** | | | | | | | | | | | | | | |
| CD44^+^ populations in CD8^+^ T cells | | *Sting^fl^*  +Vehicle | | | *Sting^fl^*  +CTLA4-Ig | | | *Lysm^Cre^Sting^fl^* + Vehicle | | *Lysm^Cre^Sting^fl^* + CTLA4-Ig | | | Two-way ANOVA test | |
|  |  | 32.7 | | | 19.6 | | | 21.5 | | 18.6 | | |  |  |
|  |  | 30.3 | | | 22.5 | | | 16.7 | | 17.5 | | |  |  |
|  |  | 27.4 | | | 18.1 | | | 15.4 | | 18.8 | | |  |  |
|  |  | 22.5 | | | 18.5 | | | 18.0 | | 20.7 | | |  |  |
|  |  | 25.6 | | | 19.4 | | | 17.6 | | 19.7 | | |  |  |
| **Figure 6E** | | | | | | | | | | | | | | |
| CD44^+^ populations in CD4^+^ T cells | | *Sting^fl^*  +Vehicle | | | *Sting^fl^*  +CTLA4-Ig | | | *Lysm^Cre^Sting^fl^* + Vehicle | | *Lysm^Cre^Sting^fl^* + CTLA4-Ig | | | Two-way ANOVA test | |
|  |  | 23.3 | | | 14.2 | | | 17.4 | | 11.6 | | |  |  |
|  |  | 25.8 | | | 19.4 | | | 19.4 | | 9.4 | | |  |  |
|  |  | 22.9 | | | 16.4 | | | 15.6 | | 10.4 | | |  |  |
|  |  | 28.5 | | | 18.4 | | | 13.0 | | 12.5 | | |  |  |
|  |  | 27.5 | | | 16.9 | | | 12.4 | | 8.5 | | |  |  |
| **Figure 6F** | | | | | | | | | | | | | | |
| Ki-67^+^ populations in CD8^+^ T cells | | *Sting^fl^*  +Vehicle | | | *Sting^fl^*  +CTLA4-Ig | | | *Lysm^Cre^Sting^fl^* + Vehicle | | *Lysm^Cre^Sting^fl^* + CTLA4-Ig | | | Two-way ANOVA test | |
|  |  | 31.7 | | | 21.2 | | | 17.3 | | 14.5 | | |  |  |
|  |  | 27.5 | | | 23.5 | | | 16.2 | | 12.5 | | |  |  |
|  |  | 23.5 | | | 16.4 | | | 18.0 | | 13.0 | | |  |  |
|  |  | 26.4 | | | 18.4 | | | 15.2 | | 11.5 | | |  |  |
|  |  | 26.7 | | | 19.5 | | | 21.1 | | 15.8 | | |  |  |
| **Figure 6G** | | | | | | | | | | | | | | |
| Ki-67^+^ populations in CD4^+^ T cells | | *Sting^fl^*  +Vehicle | | | *Sting^fl^*  +CTLA4-Ig | | | *Lysm^Cre^Sting^fl^* + Vehicle | | *Lysm^Cre^Sting^fl^* + CTLA4-Ig | | | Two-way ANOVA test | |
|  |  | 19.7 | | | 12.2 | | | 14.8 | | 9.46 | | |  |  |
|  |  | 21.4 | | | 11.6 | | | 13.5 | | 8.38 | | |  |  |
|  |  | 16.4 | | | 9.7 | | | 12.3 | | 7.92 | | |  |  |
|  |  | 19.0 | | | 12.1 | | | 14.5 | | 11.0 | | |  |  |
|  |  | 16.6 | | | 13.9 | | | 12.0 | | 9.50 | | |  |  |
| **Figure 6H** | | | | | | | | | | | | | | |
| IFN-γ^+^ populations in CD8^+^ T cells | | Sting^fl^  +Vehicle | | | Sting^fl^  +CTLA4-Ig | | | Lysm^Cre^Sting^fl^ + Vehicle | | Lysm^Cre^Sting^fl^ + CTLA4-Ig | | | Two-way ANOVA test | |
|  |  | 15.60 | | | 8.17 | | | 8.45 | | 8.35 | | |  |  |
|  |  | 17.40 | | | 11.42 | | | 12.00 | | 10.70 | | |  |  |
|  |  | 14.50 | | | 13.04 | | | 10.42 | | 11.85 | | |  |  |
|  |  | 13.50 | | | 9.75 | | | 10.04 | | 11.03 | | |  |  |
|  |  | 15.20 | | | 10.54 | | | 9.46 | | 7.98 | | |  |  |
| **Figure 6I** | | | | | | | | | | | | | | |
| FOXP3^+^ populations in CD4^+^ T cells | | *Sting^fl^*  +Vehicle | | | *Sting^fl^*  +CTLA4-Ig | | | *Lysm^Cre^Sting^fl^* + Vehicle | | *Lysm^Cre^Sting^fl^* + CTLA4-Ig | | | Two-way ANOVA test | |
|  |  | 13.7 | | | 18.7 | | | 20.6 | | 27.7 | | |  |  |
|  |  | 14.8 | | | 20.5 | | | 24.6 | | 28.4 | | |  |  |
|  |  | 15.4 | | | 23.2 | | | 18.6 | | 24.8 | | |  |  |
|  |  | 17.9 | | | 19.4 | | | 22.6 | | 26.8 | | |  |  |
|  |  | 12.4 | | | 21.7 | | | 22.7 | | 27.0 | | |  |  |
| **Figure S4D** | | | | | | | | | | | | | | |
| NOS2^+^ populations in macrophages | | | | *Sting^fl^* | | | *Lysm^Cre^Sting^fl^* | | | | Student’s *t* test  *P*=0.0005 | | | |
|  |  |  |  | 46.9 | | | 32.8 | | | |  |  |  |  |
|  |  |  |  | 46.3 | | | 42.1 | | | |  |  |  |  |
|  |  |  |  | 55.0 | | | 32.7 | | | |  |  |  |  |
|  |  |  |  | 57.1 | | | 35.2 | | | |  |  |  |  |
|  |  |  |  | 49.6 | | | 33.9 | | | |  |  |  |  |
| **Figure S4E** | | | | | | | | | | | | | | |
| MHC-II^+^ populations in macrophages | | | | *Sting^fl^* | | | *Lysm^Cre^Sting^fl^* | | | | Student’s *t* test  *P*=0.0077 | | | |
|  |  |  |  | 50.4 | | | 38.9 | | | |  |  |  |  |
|  |  |  |  | 45.0 | | | 41.5 | | | |  |  |  |  |
|  |  |  |  | 43.1 | | | 33.7 | | | |  |  |  |  |
|  |  |  |  | 52.8 | | | 39.2 | | | |  |  |  |  |
|  |  |  |  | 46.2 | | | 42.6 | | | |  |  |  |  |
| **Figure S4F** | | | | | | | | | | | | | | |
| CD80^+^ populations in macrophages | | | | *Sting^fl^* | | | *Lysm^Cre^Sting^fl^* | | | | Student’s *t* test  *P*=0.0104 | | | |
|  |  |  |  | 53.1 | | | 39.9 | | | |  |  |  |  |
|  |  |  |  | 46.3 | | | 42.1 | | | |  |  |  |  |
|  |  |  |  | 55.0 | | | 34.7 | | | |  |  |  |  |
|  |  |  |  | 57.1 | | | 47.2 | | | |  |  |  |  |
|  |  |  |  | 49.6 | | | 46.9 | | | |  |  |  |  |
| **Figure S4G** | | | | | | | | | | | | | | |
| CD86^+^ populations in macrophages | | | | *Sting^fl^* | | | *Lysm^Cre^Sting^fl^* | | | | Student’s *t* test  *P*=0.0002 | | | |
|  |  |  |  | 38.6 | | | 24.3 | | | |  |  |  |  |
|  |  |  |  | 42.1 | | | 23.0 | | | |  |  |  |  |
|  |  |  |  | 35.8 | | | 26.1 | | | |  |  |  |  |
|  |  |  |  | 37.9 | | | 29.5 | | | |  |  |  |  |
|  |  |  |  | 35.2 | | | 28.6 | | | |  |  |  |  |

**References**

1. Wang C, Xia T, Jiang K, et al. Apoptosis of the Tracheal Epithelium Can Increase the Number of Recipient Bone Marrow-Derived Myofibroblasts in Allografts and Exacerbate Obliterative Bronchiolitis After Tracheal Transplantation in Mice. *Transplantation*. 2016;100(9):1880-1888. doi:10.1097/TP.0000000000001230

2. Xu K-Y, Tong S, Wu C-Y, et al. Nlrp3 Inflammasome Inhibitor MCC950 Ameliorates Obliterative Bronchiolitis by Inhibiting Th1/Th17 Response and Promoting Treg Response After Orthotopic Tracheal Transplantation in Mice. *Transplantation*. 2020;104(6):e151-e163. doi:10.1097/TP.0000000000003208

3. Di Campli M-P, Azouz A, Assabban A, et al. The mononuclear phagocyte system contributes to fibrosis in post-transplant obliterans bronchiolitis. *Eur Respir J*. 2021;57(3)doi:10.1183/13993003.00344-2020

4. Wan J, Yang F, Tong S, Zhou T, Wang S. Triggering receptor expressed on myeloid cells-1 aggravates obliterative bronchiolitis via enhancing the proinflammatory phenotype of macrophages. *Int Immunopharmacol*. 2024;143(Pt 1):113274. doi:10.1016/j.intimp.2024.113274
